# Hidden figures: Revisiting doping prevalence estimates previously reported for two major international sport events in the context of further empirical evidence and the extant literature

**DOI:** 10.3389/fspor.2022.1017329

**Published:** 2022-12-05

**Authors:** Andrea Petróczi, Maarten Cruyff, Olivier de Hon, Dominic Sagoe, Martial Saugy

**Affiliations:** ^1^School of Life Sciences, Pharmacy and Chemistry, Faculty of Health, Science, Social Care and Education, Kingston University, London, United Kingdom; ^2^Department of Movement Sciences, Faculty of Movement and Rehabilitation Sciences, Katholieke Universiteit (KU) Leuven, Leuven, Belgium; ^3^Willibald Gebhardt Research Institute, University of Münster, Münster, Germany; ^4^Faculty of Social Sciences, Utrecht University, Utrecht, Netherlands; ^5^Doping Authority Netherlands, Capelle aan den IJssel, Netherlands; ^6^Department of Psychosocial Science, University of Bergen, Bergen, Norway; ^7^Research and Expertise in anti-Doping Sciences (REDs), Institute of Sport Sciences, University of Lausanne, Lausanne, Switzerland

**Keywords:** athlete, performance enhancement, doping, Randomised Response Technique, prevalence, Single Sample Count, prohibited substance, elite sport

## Abstract

**Background:**

High levels of admitted doping use (43.6% and 57.1%) were reported for two international sport events in 2011. Because these are frequently referenced in evaluating aspects of anti-doping, having high level of confidence in these estimates is paramount.

**Objectives:**

In this study, we present new prevalence estimates from a concurrently administered method, the Single Sample Count (SSC), and critically review the two sets of estimates in the context of other doping prevalence estimates.

**Methods:**

The survey featuring the SSC model was completed by 1,203 athletes at the 2011 World Championships in Athletics (WCA) (65.3% of all participating athletes) and 954 athletes at the 2011 Pan-Arab Games (PAG) (28.2% of all participating athletes). At WCA, athletes completed both UQM and SSC surveys in randomised order. At PAG, athletes were randomly allocated to one of the two surveys. Doping was defined as “having knowingly violated anti-doping regulations by using a prohibited substance or method.”

**Results:**

Estimates with the SSC model for 12-month doping prevalence were 21.2% (95% CI: 9.69–32.7) at WCA and 10.6% (95% CI: 1.76–19.4) at PAG. Estimated herbal, mineral, and/or vitamin supplements use was 8.57% (95% CI: 1.3–16.11) at PAG. Reliability of the estimates were confirmed with re-sampling method (*n* = 1,000, 80% of the sample). Survey non-compliance (31.90%, 95%CI: 26.28–37.52; *p* < 0.0001) was detected in the WCA data but occurred to a lesser degree at PAG (9.85%, 95% CI: 4.01–15.69, *p* = 0.0144 and 11.43%, 95% CI: 5.31–11.55, *p* = 0.0196, for doping and nutritional supplement use, respectively). A large discrepancy between those previously reported from the UQM and the prevalence rate estimated by the SSC model for the same population is evident.

**Conclusion:**

Caution in interpreting these estimates as bona fide prevalence rates is warranted. Critical appraisal of the obtained prevalence rates and triangulation with other sources are recommended over “the higher rate must be closer to the truth” heuristics. Non-compliance appears to be the Achilles heel of the indirect estimation models thus it should be routinely tested for and minimised. Further research into cognitive and behaviour aspects, including motivation for honesty, is needed to improve the ecological validity of the estimated prevalence rates.

## Overview of the doping prevalence estimations at two major sport events in 2011

Determining the prevalence of doping behaviour is a strategic priority for the World Anti-Doping Agency (WADA) and the wider anti-doping community. Without a measure for doping behaviour, reliably evaluating the effectiveness of anti-doping programs is illusive. Having robust and reliable sector-wide data on doping prevalence, which then can be segmented for sports or countries if required, can feed into risk assessments, inform testing plans, and facilitate monitoring trends over time.

In this study, doping is understood as the use of a prohibited substances and/or method. While this definition is not as comprehensive as the definition of doping under the World Anti-Doping Code (2), it captures the subset of anti-doping rule violations that could be considered most relevant for a survey of athlete behaviour (i.e., the conscious use of prohibited substances and/or methods), and for evaluating the effectiveness of anti-doping programmes. Resources and skills required for analytical methods (i.e., doping control testing from urine and blood samples) or the Athlete Biological Passport limit both the scope and timeframe of data for doping prevalence (3, 4). In contrast, the survey method is inexpensive and can theoretically cover 100% of the target population with no significant increase in costs. Among the plethora of survey options, indirect estimation models that rely on randomisation or fuzzy response techniques are promising research tools for epidemiology-level investigations of sensitive behaviour for their enhanced level of protection over and above standard anonymity options (5, 6). This protection is not only for the respondents, which tends to be the focal point in studies justifying the use of such methodology, but the researchers as well.

In 2011, the World Anti-Doping Agency (WADA) established a working group to review the available evidence for doping prevalence and to develop a new method for measuring doping prevalence in a robust, accurate and consistent manner. Specifications for this new method include being suitable for: (1) establishing doping prevalence at the population level (e.g., a specific sport worldwide or multi-sport at a given country), (2) appropriate for periodic self-assessment of doping prevalence worldwide, and (3) reasonable requirements for infrastructure to be implementable by all. Working towards this objective, and after a comprehensive review of the available options, survey-based methods from the random/fuzzy response family were selected for their ease of use, level of protection, and cost-effectiveness. The working group conducted a series of pilot studies in non-athlete populations and designed two survey formats, built on different techniques. One of these techniques was the well-established Unrelated Question Model (7). The other one was a then-newly developed model called Single Sample Count (8). The surveys based on these models were formulated and administered at two international sport events: first at the 13th International Association of Athletics Federations World Championships in Athletics (WCA) in Daegu, South Korea, then at the 12th Pan-Arab Games (PAG) in Doha, Qatar held in August and December, respectively.

Public dissemination of the results was embargoed until 2016. Despite this, partial results from these surveys, specifically estimations for past year doping use from the UQM survey were made public at various timepoints. First, these were published in a New York Times article on August 22, 2013 (9). Two years later, a previously prepared confidential manuscript was made public on the UK Parliamentary Committee website under parliamentary privilege as part of the investigation into doping in the UK (10). Following these events, the manuscript was approved by the IAAF for submission in late 2016 and finally published in Sports Medicine on August 28, 2017 (1). In this paper, the prevalence of past year doping at the two sport events were reported as 43.6% (95% CI: 39.4–47.9) at WCA and 57.1% (52.4–61.8) at PAG. Since then, the paper has been often cited as evidence for widespread doping and critiquing aspects of the current anti-doping system [e.g., (11–14)]. Results from the second survey utilising the SSC model were not published but frequency counts of the responses were presented in the Supplementary material [(1), Table 6, p. 30].

The delay in publication of the results also led to a break in the work of the Prevalence Working Group (PWG),^1^ which was then re-established in 2017 with the overarching goal to complete the survey development and create a framework for combining evidence for doping from a number of different sources such as doping control testing figures, Athlete Biological Passport and self-reports through questionnaires and surveys. As members of the current WADA PWG, we feel that making the full results from 2011 publicly available is both important and necessary. Therefore, with the support of WADA, the Athletics Integrity Unit and World Athletics, in this paper we report the results obtained *via* the SSC survey in full. We also take this opportunity to clarify some apparent assumptions and respond to the assertions contrasting surveys as simple and easy methods for establishing prevalence rates with a lack of official figures on doping prevalence. For example, Pielke (15) conflates caution justified on scientific grounds with a lack of political will and reluctance in global rollout of the survey method when writes:

Perhaps the most important result from Ulrich et al. is not that half of elite athletes are doping, but rather quantifying prevalence and how it changes over time is not just possible, but readily available. Anti-doping agencies, sport organisations and the athletes whom they oversee simply have to decide that gathering such data is a priority. So what are we waiting for? (p. 208).

Haphazard survey construction, however, can lead to unreliable data and incorrect interpretation, which may ultimately result in lack of trust in survey methodology as being “less-scientific.” Therefore, in this paper, we aim to address two objectives:

(1) First, we expand the evidence synthesis on doping prevalence by Gleaves et al. (4) and delve deeper into prevalence estimations made by indirect estimations. With this, we offer a detailed view of the indirect survey methods for estimating doping prevalence in elite sports, and draw conclusions from the collective evidence for doping prevalence.
(2) Next, we detail one specific model—the Single Sample Count (8)—which we then use for the empirical part of this study. We present detailed results from the parallel datasets from WCA and PAG in 2011, and discuss—in literature context—the plausible reasons for the observed divergence between the two estimates for the same sample by different models.

By challenging the validity of these estimations as true prevalence rates, we intend to incite meaningful and sector-wide discussions on how to obtain reliable and valid evidence for doping prevalence, catalyse research into cognitive and behavioural factors associated with randomised/fuzzy response survey formats, and foster further development in indirect estimations. We discuss the challenges associated with the indirect estimation methods, and draw attention to key areas where improvements can be made. We complete with recommendations for future method development. To facilitate further research and advance our understanding of how these models work in field settings, we offer context on how these two, widely different, prevalence estimations should be interpreted.

## Estimating doping prevalence in elite sports with indirect survey methods

The Randomised Response Technique (RRT) was originally proposed by Warner (16) to address concerns regarding self-protection and impression management *via* dishonest answering in surveys. Owing to the survey design, linking individual responses to the sensitive item (i.e., admitting anti-doping rule violation) is not possible. Prevalence of the admitted sensitive behaviour can only be estimated for the entire sample (all respondents combined). This added protection is achieved by adding a degree of uncertainty with a known probability to the responses. This added noise makes identification of the individual responses impossible. However, because the distribution of the added “noise” is known (e.g., rolling “six” on a 6D dice or a birthday falling in a given season), it allows for a sample or population level estimation of the affirmative answer to the question of interest. For example, researchers can estimate that 20% of the respondents admitted doping use but not able to pick the individual responses or respondents who constitute the 20%. As there is no way for the researcher to know how participants respond individually, these survey formats are suitable for investigations where a reporting requirement on individuals is prohibited, or research on sensitive/transgressive, illegal and/or criminal behaviour.

Over the last two decades, a total of 19 unique studies with some indirect estimation model variant to date returned doping prevalence rates between 0 and 58% in elite sport (Table 1). Among the models employed, random techniques such as the Forced Response (FR) model (38) and Unrelated Question (UQ) model (7), with or without a cheating detection extension (39), have dominated the field. The Single Sample Count (SSC) was the only non-random model utilised in estimating doping prevalence (8, 40). Characteristically, in Forced Response (FR) models and its variants (38, 41), “noise” is added to the survey question(s), which prevents the researcher from knowing whether a participant responded to the question (e.g., “did you use doping?”) or to the instructed command (e.g., “if you roll 1 or 6 on the dice, just say ‘yes' regardless of whether you used doping or not” vs. “respond honestly if you roll 3 or 4”). Still relying on the element of uncertainty for the questions, the Unrelated Question (UQ) model (7) instructs participants to answer either the sensitive target question (i.e., “Did you use doping?”) or an unrelated innocuous question (i.e., “Is your birthday in the spring?”) depending on the outcome of a randomisation exercise (i.e., rolling a dice, or using a birthday, specified digits of a phone number or banknote serial number). In contrast, in the Single Sample Count (SSC) model (8, 40), the protective “noise” is added to the response format, not to the question. Application of the SSC model includes assessing the prevalence of the use of neuroenhancements (42), abortion (43), drinking among first year university students (44, 45) and illegal killing of wildlife (46). Each of these variants has advantages and disadvantages in terms of effectiveness, efficiency, as well as factors influencing compliance with the survey instructions such as understanding, cognitive demand and trust (6, 47–52).

**Table 1:** Summary of studies estimating doping prevalence in elite sport using indirect (randomised and non-random) estimation models.

**Study type**	**Timeframe**	**Range of estimated prevalence of doping**	**Range of estimated non-compliance**	**Models**	**References**
Published research studies	Lifetime/ever	0.0–26.7%	3.1–34.4%	Unrelated question; Randomised response; Forced response	(17–25)^*^
	Past 12 months/Last season/current season	0.0–57.1%	19.0–31.3%	Unrelated question; Forced response	(1, 17, 19, 21–30)^*^
Commissioned targeted studies	Past 3 months	0.0–9.2%	6.7–7.6%	Single Sample Count	(31)
	Past 12 months	3.15–9.2%	1.8% (SSC) Not considered (FR/Kuk)	Single Sample Count; Forced response (FR)/Kuk design	(32–34)
Unpublished studies		5.9–32.7% (athletes) 58.0% (bodybuilders)	24.5–40.7% (athletes) 6.5% (bodybuilders)	Forced response	(35–37)

Asterisk (*) marks identical data, or identical study published in German and English.

Although direct comparison of these estimates is hindered by the lack of uniform definition of “doping use” or comparable timeframe, the bulk of the evidence appears to estimate the rate of admitted doping use between 0 and 25%. The high global estimate of close to 50% of athletes affirming doping use by Ulrich et al. (1) do not coincide with other prevalence estimates except those obtained for competitive bodybuilders at 58% (36, 37). A recent study (53) re-analysed the data from Ulrich et al. (1) to demonstrate the advantage of curtailed sampling and offered confidence for such method to establish that doping prevalence is higher than 10%, an arbitrary threshold which is a considerable reduction of the initially reported 43.6% and 57.1% for the two major sport events in 2011. Notably, this method does not aim to estimate doping prevalence, only to find evidence that doping prevalence is higher than a set threshold.

Non-compliance was only considered in nine studies. Collectively, these estimates showed that a considerable proportion of athletes up to 40.7% do not comply with the survey instructions. Studies variably dealt with this segment, with some adding the percentage of estimated non-compliance to “honest dopers” to derive a possible upper estimate (17, 19, 21, 22, 54), whereas others reporting non-compliance separately (35–37), or allocating only a proportion of the non-compliant segment to “doping prevalence” (31, 32). Notwithstanding the reasons for non-compliance, its magnitude should raise an alarm about a potentially significant distorting effect in prevalence estimations obtained *via* indirect models.

As different prevalence estimations for unique samples were obtained *via* different estimation models, it is impossible to find out whether the observed differences are due to either the sample characteristics or method differences, or the combined effect of both factors. A recent systematic review and evidence synthesis of the available evidence for doping prevalence revealed the limiting impact of methodical inconsistency and varying quality of data reporting (4). Although data obtained *via* indirect estimation models generally scored high on quality assessment (average: 63.0% with half of the included studies scoring in the upper 75%), they were not free of limitations in terms of comparability and generalizability.

The “higher must be better” assumption (55), which is widely observed and criticised outside the doping context (56), appears to be present in doping prevalence studies. However, in most cases, the empirically obtained prevalence estimates have been compared to the “official” WADA laboratory reports of Adverse and Atypical Analytical Findings. Two earlier studies (18, 36, 37) compared estimated prevalence with results obtained *via* direct questioning, both of which confirmed the expected pattern of higher prevalence *via* the more protective indirect methods than direct questioning. Specifically, Plessner and Much (36, 37) reported 7.8% prevalence through direct questioning (10.6% for bodybuilders and 5.5% for other athletes), whereas the indirect estimation of admitted doping was considerably higher at 42.5% (58.0% for bodybuilders and 32.7% for other athletes). In the study by Striegel et al. (18) among German elite athletes, doping prevalence figures from official doping testing (0.81% for German athletes) was contrasted with admitted doping in direct questioning (0.2%) and obtained *via* indirect estimation (6.8%). Due to the research design, participants were randomly allocated to the direct question model or the indirect estimation model. The difference in admitting doping was not observed for illegal drug use (7% admitted under both conditions).

With a few notable exceptions e.g., (26, 43) and the empirical section of the present study, no previous studies compared different models or attempted to validate the obtained estimates. James et al. (26) compared two indirect estimations with a third indirect method known as the Network Scale-Up [NSU; (57)] where respondents are asked to quantify their social network and report the exact number of doping users within. Additionally, participants completed all formats with the two indirect methods (UQ and SSC) appearing in random order. Whilst there was no significant difference in admitting dietary supplements with hormonal boosting effects (SSC: 62.6%, UOQ: 59.4%), the different methods yielded large different estimates for doping use in the past 12 months: SSC: 12.8%; UQ: 55.4%; NSU known: 3.9% and NSU suspected: 21.4%. This study is illuminating in many ways: it replicated the research design used at the WCA in 2011, triangulated the results with a third method, and offered a tentative explanation for the observed differences, thereby contributed to subsequent model development.

## Empirical analysis of the SSC data from WCA and PAG, 2011

In this section, we aim to integrate the initial aim of these two studies namely developing a reliable survey-based method for estimating doping prevalence globally. The overall survey setup, general conditions for data collection and procedures are detailed in Ulrich et al.'s report (1). In the following sections, we limit the description to the SSC survey format. Detailed description of the SSC model is presented in Supplementary material 1.

### Measures

At WCA and PAG, the doping prevalence measures comprised a single statement: “*I have knowingly violated anti-doping regulations by using a prohibited substance or method in the past 12 months*.” Dietary supplement use was assessed at PAG with a single question: “*I have used herbal, mineral, and/or vitamin supplements in the last 12 months*.” Both target questions were incorporated into four unrelated and innocuous statements which asked about a respondent-selected person's birthday confirming to *Bernoulli* distribution (*p* = 0.5). The respondents were instructed to think of a random person whose birthday they know and keep this person in mind when answering the survey question, without disclosing which person (or what birth date) this was. This way, only the respondents had full information about the innocuous questions, the researcher could not tell or guess the responses to these questions/statements. The exact wording of the survey questions is shown in Table 2.

**Table 2:** SSC survey design.

**Sport event**	**Question set**	**Items**
WCA	Doping set	1. The person's date of birth falls between July and December inclusive.
		2. The person's date of birth is in February, April, June, August, October, or December.
		3. I have knowingly violated anti-doping regulations by using a prohibited substance or method in the past 12 months.
		4. My own date of birth falls between July and December (inclusive).
		5. own date of birth is in February, April, June, August, October, or December.
PAG	Doping set	1. The birthday of the person I am thinking of falls in the second half of the year (July–December).
		2. The birthday of the person I am thinking of is in February, April, June, August, October, or December.
		3. The birthday of the person I am thinking of falls in the first half of the month (1–15 inclusively).
		4. The birthday of the person I am thinking of is on an odd day (on or ending with 1, 3, 5, 7, 9).
		5. I have knowingly violated anti-doping regulations by using a prohibited substance or method in the past 12 months.
	Dietary supplement set (instructed to think of a different person)	1. The birthday of the person I am thinking of falls in the first half of the year (January–June).
		2. The birthday of the person I am thinking of is in February, April, June, August, October, or December.
		3. The birthday of the person I am thinking of falls in the first half of the month (1–15 inclusively).
		4. The birthday of the person I am thinking of is on an even day (on or ending with 0, 2, 4, 6, 8).
		5. I used herbal, mineral or vitamin supplements in the last 12 months.

The position of the target (sensitive) question was fixed in the first study (WCA) to the middle position. The order was randomised in the second study (PAG). Response options in all sets were: “0 or 5,” “1,” “2,” “3,” or “4” yes responses in total. Two out of the four innocuous questions are negated in the second set. Thus, theoretically, there should be no way of inferring the answer to the sensitive question without knowing the exact birthday. Nevertheless, it might be the case that the athletes read the questions hastily and do not notice the difference. To mitigate this possibility, we instructed respondents to think of a different person for the second set.

The survey in the WCA study was available in 21 languages, based on the list of languages officially used by the IAAF. Owing to the more homogeneous sample, the PAG survey was only made available in three languages (English, French, and Arabic).

As athletes in the WCA study completed both models, we asked participating athletes to indicate which one of the two models they preferred. The question “*which one of the two versions did you prefer?*” was followed by two response options: “the one I answered with a yes or no” and “the one I answered with a number.”

Completion time and language were recorded at both events. Participants were also asked to identify their sport in the PAG study but without specifying their disciplines. Disciplines within athletics at WCA were not recorded.

### Participants

At the WCA in Daegu, South Korea, 1,203 athletes agreed to complete the survey (65.3% of all participating athletes) of which 50.1% (*n* = 603) received the SSC model first. The highest number of participating athletes completed the survey in English (42.2%), followed by Spanish (11.5%), Russian (9.5%), French (9.1%), with the remaining 27.7% comprised of 17 languages together [data obtained from (1), Supplementary material 2].

At the PAG in Doha, Qatar, 1,910 athletes agreed to participate (57.1% of all participating athletes). Of these, 945 athletes received the SSC survey format (49.8% of the sample and 28.2% of the athlete population at PAG). Majority of the athletes completed the survey in Arabic (77.1%). Athletes represented 27 sports with athletics being the only sport with a >10% share (14.1%) of the sample. Of the other 26 sports, between 5 and 10% were basketball, football, handball, shooting, table tennis, taekwondo, and volleyball. The remaining sports (< 5% each) were aquatics, archery, bodybuilding, bowling, boxing, chess, cue sports, cycling, fencing goalball golf gymnastics judo karate, paralympic sports, sailing, squash, weightlifting, and wrestling. No other demographic data were recorded.

### Procedure

#### Data collection

As described in Ulrich et al. (1), SSC and UQM were tested and compared in two experimental designs. The first study took place during the WCA in Daegu, South Korea. This sample represents a single sport with multi events, where many disciplines are considered to be high risk for doping. In total, 1,841 athletes participated at the event. Data collection took place over 10 days between August 25 and September 3, 2011. In the second study at PAG in Doha, Qatar, data collection took place over 4 days between December 10 and 13, 2011. It was a multisport event with 3,346 accredited athletes. Owing to the nature of the event, this sample was more diverse in terms of sports represented but less diverse in terms of culture, faith, and ethnicity than the sample in the WCA study.

At WCA, respondents answered the same question on doping use presented in both SSC and UQM formats. The order of the survey variants was randomly allocated. At the end of the survey, respondents were asked to indicate a preference for one of the two formats. Data were collected in social places (i.e., dining hall, recreational area, and outreach activity area) in the respective Athlete villages.

At PAG, respondents were randomly allocated to complete either the SSC or the UQM survey. This time, respondents were asked to answer two questions: one sensitive question (doping use, identical to the question used at WCA) and one less sensitive but comparable question (dietary supplement use). To ensure an equal split, the software detected the proportion of completed UQM and SSC on each iPad individually and presented the version to the new respondent that had less. In the case of the first or exactly 50/50% split, the allocation was random. The doping and supplement questions were presented in the same format (i.e., UQM or SSC) in randomised order.

#### Equipment

Data collection was exclusively electronical, using volunteers and iPads equipped with a custom-made software. Camera access was physically blocked on all iPads. Data were stored locally and periodically submitted to a designated secure online database.

#### Consent

Athletes were informed that the primary aim of the study was to test a unique survey format. Participation was voluntary after informed consent. Consent was implied by active participation (completing and submitting the survey). Athletes could withdraw from the study during data collection by aborting the survey but retrospectively removing data was not possible. The study was favourably reviewed by the Faculty Research Ethics Committee, Faculty of Science, Engineering and Computing, Kingston University.

### Data

To allow comparison, we work with the frequency counts for the SSC response options as reported in Ulrich et al.'s (1) Supplementary material 1 (Table 6, p. 30). The frequency counts (raw data) are displayed in Table 3.

**Table 3:** Frequency counts of the SSC response options obtained at the two sport events (WCA and PAG) in 2011, alongside the UQM method.

**Target question**	**Design**	**Response options**	**WCA (*****n*** = **1,203)**			**PAG (*****n*** = **954)**		
			**1st**	**2nd**	**Total**	**1st**	**2nd**	**Total**
Doping	Same sample UQM and SSC presented in random order	0 or 5	78	76	154	40	37	77
		1	169	164	333	134	140	274
		2	211	215	426	139	169	308
		3	95	90	185	97	115	212
		4	50	55	105	45	38	83
Total			603	600	1,203	455	499	954
Supplement	Separate sample Questions presented in random order	0 or 5	-	-	-	33	43	76
		1	-	-	-	152	118	270
		2	-	-	-	175	167	342
		3	-	-	-	99	83	182
		4	-	-	-	40	44	84
Total						499	455	954

### Data analysis

Raw individual level data are not required for estimating the proportion of athletes who answered the doping and/or dietary supplement question(s) affirmatively. To estimate prevalence, we only need to know how many athletes in the sample selected each response option. These frequency counts are given in Table 3.

Shapiro-Wilk test with Lillefors significance correction was used for testing normality. Association between order and survey preference at WCA was tested using chi-square test of association with Yates correction, and Fisher exact *p* for significance. Order effect of response times was tested using independent *t-*test (WCA) and mixed model ANOVA (PAG).

Prevalence estimations were calculated using the expectation maximisation (EM) method (58). Akaike Information Criterion (AIC) and Bayesian Information Criterion (BIC) were also calculated for each hypothesis to inform the decision about model fit. Estimation ranges are expressed as 95% confidence intervals. Reliability of the estimated parameters for each hypothesis was estimated by a re-sampling procedure with 1,000 artificial response sets and 80% of the empirical data using the full respective datasets. Conditional probabilities were also calculated for each response option based on the estimated prevalence rate. That is, a probability of the response containing an affirmative answer to the sensitive (doping) question was calculated for each response options in each sample.

### Results

In the data collected at WCA, where the sensitive doping question was always presented in the middle of the list (as third statement), the order when the SSC model was presented made no impact on the response distribution (*X*^2^ = 0.504, *p* = 0.973).

At PAG, the questions about the supplements and prohibited methods and/or substances were presented in random order (see Table 1). The order had no impact on the pattern of responses (*X*^2^ = 5.744, *p* = 0.220).

Frequency distribution of “0 or 5,” “1,” “2,” “3,” or “4” affirmative responses significantly diverged from normal distribution [WCA: *W*_(1, 203)_ = 0.911, *p* < 0.001, PAG doping: *W*_(954)_ = 0.914, *p* < 0.001; PAG supplements: *W*_(954)_ = 0.911, *p* < 0.001].

#### Estimated prevalence of doping and nutritional supplement use at 2011 PAG using the SSC survey data

##### Non-compliance

Non-compliance detection in the SSC model is linked to the proportion of the “0 or 5” response option. The expected probability (*p*) for this response option is 1/16 and it is independent of the admitted doping prevalence *d* (40). In other words, if respondents are compliant with the survey instruction and respond honestly, regardless of whether they used doping or not, *p* = 0.0625 is expected for the “0 or 5” option. A statistically significant deviation in the observed data from this expected *p* is the evidence for non-compliance. Testing for non-compliance (H0: no sign of non-compliance) showed statistically significant evidence for non-compliance (*p* < 0.0001).

##### Doping prevalence estimation

All considered, the best fitting model—based on the highest Log-likelihood value and the smallest AIC and BIC values—was H4. Detailed results are shown in Table 4.

**Table 4:** Estimated use of prohibited performance enhancing substances and/or methods at WCA (12-month prevalence).

	**Model fit**			**Estimated % of admitted doping (*d*)**	**Estimated % of non-compliance (*nc*)**
**H**	**Log-likelihood**	**AIC**	**BIC**		***p =* 1.1.E−16**
H0	−1,854.0342	3,708.07	3,708.07		
H1	−1,853.9311	3,709.86	3,714.95	0.0132 ± 0.0576	0
H2	−1,819.0675	3,642.14	3,652.32	0	0.3031 ± 0.0800
H3	−1,819.5104	3,643.02	3,653.21	0.0132 ± 0.0576	0.0699 ± 0.0201
**H4**	**−1,799.2650**	**3,602.53**	**3,612.72**	**0.2124** **±0.0873**	**0.3190** ±**0.0562**
H5	−1,853.9311	3,711.86	3,722.05	0.0132 ± 0.0576	0

Bold denotes the best fitting model.

H4 characteristically assumes that non-compliant respondents select randomly from the lower half of the response options, specifically selecting “0 or 5,” “1,” or “2” regardless of the true response. Intuitively, it also feels like a “safe” option. Under this hypothesis, 21.24% is estimated to have admitted using prohibited substances and/or methods, and 31.9% are assumed to be non-compliant meaning that 7.27 ± 3.98% are guilty non-compliers (i.e., are involved in doping and non-compliant), 14.96 ± 7.14% are involved AND compliant, 25.62 ± 7.21% are innocent non-compliers (i.e., are non-compliant but not involved in doping), and 54.12 ± 10.37% are not involved AND compliant.

The conditional probability of being guilty of doping for each response, assuming the model parameters fitted above, was established as follows: with a response “0 or 5,” *p* for affirmative answer to the sensitive doping question is 0.2124; response “1” *p* = 0.1268; response “2” *p* = 0.171; response “3” *p* = 0.2881, and response “4” *p* = 0.519.

A detailed method for estimating prevalence and non-compliance with the SSC model is presented in Nepusz et al. (40). Here, we only offer a worked example for the numerical calculation of the estimated doping prevalence rate under H4. The SSC-EM estimation model estimates the probability of the presence of the sensitive attribute (*D*) and the proportion of non-compliance (*NC*). From these estimations, assuming that *D* and *NC* are independent, we can attribute non-compliance to those with the sensitive attribute (guilty non-compliance) and to those who do not carry this attribute (innocent non-compliance) as set in Equations 1 and 2.


(1) $$\begin{array}{l} D=\left(d\times nc\right)+\left(d\times \left(1-nc\right)\right) \end{array}$$

(2) NC= ((1−d)×nc)+(d×nc)

The remaining proportion of the estimated probability then belongs to the compliant non-carriers of the sensitive attribute (Equation 3), denoted by H.


(3) H=(1−d)×(1−nc)

To attribute non-compliance, we calculate the probabilities of each combination of compliance and attribute. Thus, the estimated overall probability of NC can be divided into innocent non-compliance (Equation 4) and guilty non-compliance (Equation 5). Equally, D can be divided into the probabilities of the honestly admitted sensitive attribute (Equation 6) and the guilty non-compliance denying the sensitive attribute (Equation 7). To obtain probabilities for each combination of compliance and attribute, we calculate the lowest (min) and highest (max) probability values for each combination independently, then take the midpoint to receive the probability estimate for each, before adding them accordingly:


(4) $$\begin{array}{l} \left(1-d\right)\times nc\\ =\frac{\left(\left(1-d_{\min}\right)\times nc_{\max}\right)+\left(\left(1-d_{\max}\right)\times nc_{\min}\right)}{2} \end{array}$$

(5) $$\begin{array}{l} \left(1-d\right)\times \left(1-c\right)\\ =\;\frac{\left(\left(1-d_{\min}\right)\times \left(1-c_{\min}\right)\right)+\left(\left(1-d_{\max}\right)\times \left(1-c_{\max}\right)\right)\;}{2} \end{array}$$

(6) $$d\times \left(1-c\right)=\frac{\left(d_{\min}\times \left(1-c_{\max}\right)\right)+\left(d_{\max}\times \left(1-c_{\min}\right)\right)}{2}$$

(7) $$\begin{array}{l} d\times c=\;\frac{\left(d_{\min}\times \;c_{\min}\right)+\left(d_{\max}\times c_{\max}\right)}{2} \end{array}$$

The values for the estimated prevalence of the sensitive behaviour (*D*) and non-compliance (*C*) are obtained from the SSC-MLE selecting the combination of *D* and *C* that fits the observed variance in the empirical data the best. Under H4 (Table 3), the probability of doping use (*D*) is 0.2124 ± 0.0873 whereas the probability of non-compliance with survey instructions (*C*) is 0.3190 ± 0.0562. Therefore, with 95% probability, the value of *D* is between 0.1251 and 0.2997, and the value of *C* is between 0.2628 and 0.3752. Assuming that the CIs are symmetrical, we can take the midpoint between the lowest and highest values to derive each of the four components of the sensitive attribute (*d*) and non-compliance (*nc*) matrix:


$$\begin{array}{l} d\times \left(1-nc\right)\\ =\frac{\left(0.1251\times \left(1-0.3752\right)\right)+\left(0.3752\times \left(1-0.2628\right)\right)}{2}\\ =\frac{0.07816+0.0.2209}{2}=0.1496\\ d\times nc=\frac{\left(0.1251\times 0.2628\right)+\left(0.2997\times 0.3752\right)}{2}\\ =\frac{0.0329+0.1124}{2}=0.0727\\ \left(1-d\right)\times nc\\ =\frac{\left(\left(1-0.1251\right)\times 0.3752\right)+\left(\left(1-0.2997\right)\times 0.2628\right)}{2}\\ =\frac{0.3283+0.1840}{2}=0.2562\\ \left(1-d\right)\times \left(1-nc\right)\\ =\;\frac{\left(\left(1-0.1251\right)\times \left(1-0.2628\right)\right)+\left(\left(1-0.2997\right)\times \left(1-0.3752\right)\right)\;}{2}\\ =\frac{0.4375+0.6450}{2}=0.5413 \end{array}$$

Using the values for each of the four components of the sensitive attribute and non-compliance matrix, we can then calculate the non-compliance adjusted probability of the sensitive attribute (*D*), the overall probability of non-compliance (*NC*) which consists of the innocent (0.2562) and guilty (0.0727) non-compliance.


$$\begin{array}{l} D=0.0727+0.1496=\;0.2223\\ NC=\;0.2562+0.0727=0.3289\\ H=\;0.5413 \end{array}$$

The remaining 0.5413 is the probability estimate for the compliant non-dopers (*H*), which is essentially the difference between 1 and the estimated non-compliance adjusted *D* and the innocent non-compliance [(1-*d*) × *nc*]. See Supplementary material 2 for further details.

##### Dependent vs. independent non-compliance

All hypothesised models for non-compliance assume that non-compliance is independent of being guilty of doping, and attributes the estimated non-compliance with the same ratio as the estimated proportion of dopers and non-dopers in the sample. It can be argued that non-compliance to the survey instructions is not independent of the sensitive attribute (i.e., having engaged in doping), and therefore assume that the there is a higher level of non-compliance among those who used doping substances because a segment of non-compliance results from innocent non-compliance (to the same degree it is present among non-dopers) plus the motivated non-compliance due to lying about doping. Although theoretically it would be possible to model additional parameters reflecting the probability of non-compliance if one engaged in doping (*nc*1) or not (*nc*2), as well as the probability of doping (*d*), it could result in overfitting of the model with the available degrees of freedom from the 4+1 model used in this study. Specifically, in the 4+1 SSC model, the prediction model has two “real” parameters (*d*: the doping prevalence, and *nc*: the probability of non-compliance), and at least one additional degree of freedom from the hypothesised method of non-compliance, resulting in minimum 3 degrees of freedom in our hypothesised model. The input model (SSC) has only 4 degrees of freedom because the fifth fraction is determined by the other four since they must add up to 100%. Therefore, the degrees of freedom of the prediction model (*df* = 3) and the degrees of freedom of the input model (*df* = 4) being close could result in a danger of overfitting the model. To counterbalance this to a degree, we used AIC/BIC, both of which penalise for complexity, along with the likelihood values to select the best model.

Furthermore, Nepusz et al. (40) showed that the log likelihood value for *d – nc1 – nc2* can only match but not exceed the value obtained for the independent *d - c* solution. In practice, this means that even if dependence is modelled with *c1* and *c2*, the best achievable outcome is having two equally possible solutions involving different parameters but with no further guide to which one is closer to the true combination of reportedly guilty of doping and innocent, impacted upon motivated or non-motivated non-compliance. For a detailed discussion, refer to Nepusz et al. (40). In the next section, we offer an illustration through a hypothetical scenario with the WCA data.

As an illustrative example, we use model H4 which is the best fitting model from Table 4. Based on the previous results, we make the following assumptions: 15% of the respondents admits doping (thus compliant with the survey) and 55% are clean and compliant. The remaining 30% of the total respondents are non-compliant with the survey instructions but the proportion of dopers and clean athletes in this segment as well as the reasons for non-compliance are unknown. Of the estimated 30% non-compliance, any combination of motivated and non-motivated non-compliance is possible. Non-motivated non-compliance (*c*_0_), which can arise from reasons other than lying (e.g., lack of attention, misunderstanding, and language barriers), is assumed to present equally among dopers and clean athletes. Motivated non-compliance (*c*_1_) which only relates to hiding doping or avoid the perception of doping (i.e., an athlete not wanting to give the false impression of a doper because of the high number of affirmative answers to the innocuous questions, either to protect herself/himself, their country, and/or their sport), thus deliberately responding in a self-protective manner.

The results of this exercise are summarised in Table 5. Hypotheses 6–9 assume that the probability of non-compliance might be different between those who are involved in the sensitive behaviour and those who are not involved. Specifically, *Hypothesis 6* assumes that doping is present with probability *d* and respondents randomly select responses with different probabilities (pairs with H2 in Table 3 at the conceptual level). *Hypothesis* 7 describes a scenario where doping is present in the sample with probability *d* and some respondents chose the smallest possible answer instead of responding honestly (pairs with H3). *Hypothesis 8* presumes that the sensitive behaviour is present in the sample with probability *d* and some respondents chose a response randomly from the lower half of the response range by selecting “0 or 5,” “1,” or “2” instead of responding honestly (pairs with H4). *Hypothesis 9* assumes that doping is present in the sample with probability d and presents the unlikely scenario that some respondents chose a response randomly from the upper half of the response range (“3” or “4”) instead of responding honestly (pairs with H5).

**Table 5:** Doping prevalence estimation using a dependent model for non-compliance.

**H**	**Model fit**			**Estimated % of admitted doping (*d*)**	**Estimated % of non-compliance (*nc_1_*)**	**Estimated % of non-compliance (*nc*_0_)**

	**Log-likelihood**	**AIC**	**BIC**			
**H4**	**−1,799.2650**	**3,602.53**	**3,612.72**	**0.2124** **±0.0873**	**0.3190** **±0.0562**	
H6	−1,819.0676	3,642.14	3,652.32	0.0046 ± 9.1143	1.0	0.3
H7	−1,935.2124	3,874.42	3,884.61	0.2092 ± 0.0595	0.0	0.3
**H8**	**−1,799.2650**	**3,602.55**	**3,612.72**	**0.2337** **±0.1013**	**0.3811** **±0.1528**	**0.3**
H9	−2,012.3438	4,928.69	4,038.87	0.0	0.7672 ±∞	0.3
H6	−1,819.0675	3,642.14	3,652.32	0.0	0.3	0.3031 ± 0.0800
H7	−1,819.5104	3,643.02	3,653.21	0.0175 ± 0.0570	0.3	0.0658 ± 0.0205
**H8**	**−1,799.2650**	**3,602.53**	**3,612.72**	**0.2076** **±0.0837**	**0.3**	**0.3239** **±0.0796**
H9	−1,953.9962	3,711.99	3,722.18	0.0058 ± 0.0417	0.3	0.0

Bold denotes the best fitting model.

As Table 5 shows, there is always a pair of models for the same hypothesis that fit equally well (as judged by the identical maximum log likelihood values and AIC/BIC indices) but return two different outcomes for doping and non-compliance. In the present case, the difference in the estimated prevalence rates is not large but still presents a degree of uncertainty if the “real” admitted rate of doping is 21% with a range of 12–30%, or 23% with a range of 13–33%. It is also notable that even if allocation of the estimated non-compliance to exclusively those involved in doping vs. exclusively to those who are not involved, the best fitting models still suggest almost the same rate of non-compliance for the other “segment.” Additionally, no “dependent model” performed better than the best independent model (H4 in Table 4). This shows that no dependent model can outperform the independent model but introduces a degree of uncertainty that cannot be dealt with mathematically. That is, based on model fitting statistics we cannot tell which solution has more ecological validity. Unless there are external criteria to assist the selection, dependent modelling is a futile exercise. Because of the increased uncertainty and complexity, the rule of Occam's razor favouring the simpler solution should prevail.

##### Reliability of the estimated parameters

Reliability of the estimated parameters *via* resampling showed close alignment to at least one, in most cases two decimals with one exception. Resampling analysis indicated a slightly different doping prevalence estimation for H2 (*d* = 0, *nc* = 0.3036). Whilst the estimated rate of non-compliance remained at 30%, the estimated doping prevalence rate dropped to zero signalling the instability of this specific model which also was the one that returned the highest, and in the context of the reanalysis, questionable doping prevalence estimation at 30%. Results are provided in Supplementary material 3, Table 1.

#### Estimated prevalence of doping and nutritional supplement use at 2011 PAG using the SSC survey data

At PAG, each survey contained two questions. The first asked about the use of prohibited substances and/or methods (engagement in “doping” practises) and the other one asked about the use of herbal, mineral, and/or vitamin supplements in the 12 months prior to data collection.

##### Doping prevalence estimation

Testing for non-compliance (*p* = 0.0144) only showed no statistically significant evidence for non-compliance at the 0.05 level but not at the set 0.01 level.

Results presented in Table 6 suggests that H2 is the best fitting model. However, this model assumes that doping is not present in the sample but also suggests that up to one in four of the respondents were non-compliant. Due to the total absence of admitted doping is an unlikely scenario and the close to non-significant results from the cheating detection, we discarded this solution and moved to the next best option which, from the behavioural point of view, is also in line with the previous study. Similar to the results from WCA, H4 returned the second-best fitting model. This model reflects the hypothesised case where non-compliant respondents select “0 or 5,” or “1” or “2,” regardless of the true response. Intuitively, it also feels like a “safe” option. Estimations obtained from this model suggest that 10.63% (± 7.73%) of the surveyed athletes admitted doping. Non-compliance was estimated at a relatively low level at 9.85% (± 5.84%). Specifically, 1.50% ± 1.38% are guilty non-compliers (i.e., are involved in doping and non-compliant), 10.03% ± 7.59% are involved AND compliant, 9.26% ± 5.98% are innocent non-compliers (i.e., are non-compliant but not involved in doping), and 81.02 ± 12.19% are not involved AND compliant. Taking everything into account, H4 offered the best fitting estimation model. See Supplementary material 2 for further details.

**Table 6:** Estimated use of prohibited performance enhancing substances and/or methods at PAG (12-month prevalence).

	**Model fit**			**Estimated % of admitted doping (*d*)**	**Estimated % of non-compliance (*c*)**
**H**	**Log-likelihood**	**AIC**	**BIC**		
H0	−1,419.4487	2,838.90	2,838.90	-	-
H1	−1,418.2603	2,840.52	2,850.24	0.0524 ± 0.0688	0
H2	−1,410.8820	2,825.76	2,835.49	0	0.1690 ± 0.0867
H3	−1,415.7742	2,835.55	2,845.27	0.0525 ± 0.0688	0.0194 ± 0.0184
**H4**	**−1,413.1277**	**2,830.26**	**2,839.98**	**0.1063** ±**0.0773**	**0.0985** ±**0.0584**
H5	−1,418.2603	2,840.52	2,850.24	0.0523 ± 0.0688	0

Bold denotes the best fitting model.

The conditional probability of being guilty of doping for each response, assuming the model parameters fitted above, was established as follows: with a response “0 or 5,” *p* for affirmative answer to the sensitive doping question is 0.1063, response “1” *p* = 0.0394, response “2” *p* = 0.0765, response “3” *p* = 0.1514, and response “4” *p* = 0.3223.

##### Nutritional supplement use prevalence estimation

Similar to the doping question, testing for non-compliance (*p* = 0.0196) only showed statistically significant evidence for non-compliance at 0.05 level but not at the set 0.01 level. H4 also indicated relatively good model fit (Table 7). Thus, all considered, it is assumed that H4 presents a more realistic and ecologically valid scenario which is consistent with the previously observed behavioural pattern for non-compliance by selecting a low number.

**Table 7:** Estimated use of herbal, mineral and/or vitamin supplements at PAG (12-month prevalence).

	**Model fit**			**Estimated % of admitted supplement use (*d*)**	**Estimated % of non-compliance (*nc*)**
**H**	**Log-likelihood**	**AIC**	**BIC**		
H0	−1,405.6629	2,811.33	2,811.33	-	-
H1	−1,405.2946	2,812.59	2,817.45	0.0282 ± 0.0659	0
H2	−1,398.6209	2,801.24	2,810.96	0	0.1498 ± 0.0841
H3	−1,403.0767	2,810.15	2,819.87	0.0284 ± 0.0659	0.0183 ± 0.0183
**H4**	**−1,398.8982**	**2,801.78**	**2,811.52**	**0.0857** ±**0.0754**	**0.1143** ±**0.0612**
H5	−1,405.218	2,814.44	2,824.16	0	0.0223 ± 0.0476

Bold denotes the best fitting model.

Estimations of nutritional supplement use are consistent with the observed pattern for prohibited substances and methods. Model H4 performed the best mathematically but returned unrealistic data with no reported supplement use for the previous 12-month period, and the non-compliance rate holding the same. Under this hypothesis, 8.57% (± 7.54%) is estimated to have admitted using the specified type of supplements, and 11.43% (± 6.12%) are assumed to be non-compliant. This indicates that 1.44 ± 1.39% were guilty non-compliers (i.e., using nutritional supplement and non-compliant), 8.05 ± 7.20% were involved AND compliant, 10.91 ± 6.46% are innocent non-compliers (i.e., are non-compliant but not using supplements) and 81.45 ± 12.27% were not involved AND compliant. See Supplementary material 2 for further details.

The conditional probability of carrying the attribute (i.e., being a nutritional supplement user) for each response, assuming the model parameters fitted as above was established as follows: with a response “0 or 5,” *p* for affirmative answer to the sensitive doping question is 0.0857, response “1” *p* = 0.0326, response “2” *p* = 0.0616, response “3” *p* = 0.1232, and response “4” *p* = 0.2726.

##### Reliability

Reliability of the estimated parameters with re-sampling method shows identical results up to at least one decimal (see Supplementary material 3 for details).

#### Preference

Athletes at WCA reported preference for the UQM model with 63.9% favouring the UQM model (Table 8). In addition, the order in which athletes completed the survey formats showed an impact on preference [$\chi_{\left(1\right)}^{2}$ = 8.333, Fisher's exact *p* = 0.0033]. As expected, athletes exhibited a tendency for liking the model they completed second, and thus closer to the preference question.

**Table 8:** Interaction between preference for and order of the estimation models.

**Count**		**Preference**		
		**SSC**	**UQM**	**Total**
Order: First presented	SSC	193 (16.04%)	410 (34.08%)	603 (50.12%)
	UQM	241 (20.03%)	359 (29.84%)	600 (49.87%)
Total		434 (36.07%)	769 (63.92%)	1,203 (100.0%)

#### Response times

**Figure 1:**
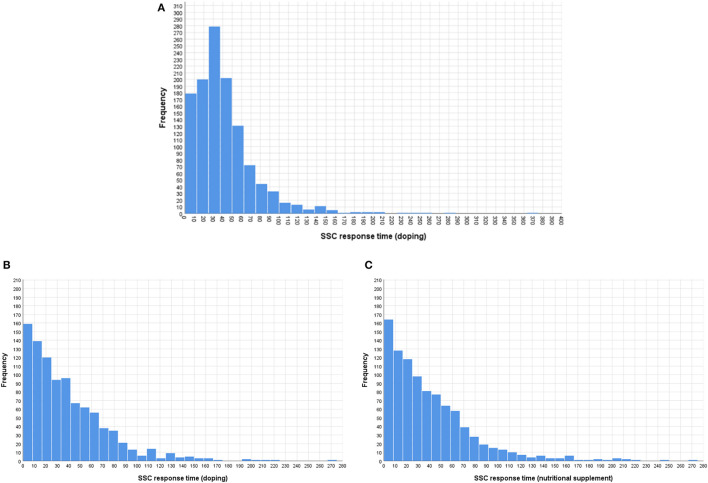
Response time distributions at WCA [**(A)** doping question] and PAG [**(B)** doping and **(C)** herbal, mineral, and/or vitamin supplements questions].

Due to the electronic data collection, we were able to measure the response time for each major survey segment (e.g., introduction, survey format, and/or survey question). The distribution of the completion times is shown in Figure 1. For the most, competing the SSC survey (excluding reading the introduction) took < 1 min. The completion time for the SSC doping question at PAG is in range with the times we measured at WCA, averaging between 38 and 45 ms despite the observed difference in non-compliance between the two events.

Contrary to response contents, the order by which the UQM and SSC models were presented at WCA had a small but statistically significant impact on the time athletes spent on completing the survey [*t*_(1, 187.8)_ = −3.581, *p* < 0.001]. Even if the UQM and SSC models were different with instructions unique to the respective model, athletes completed the SSC survey faster when it was presented after the UQM model (Mean response time = 38.29 ms, *SD* = 32.26 ms) compared to when it was presented first (Mean response time = 45.35 ms, *SD* = 36.03 ms).

**Figure 2:**
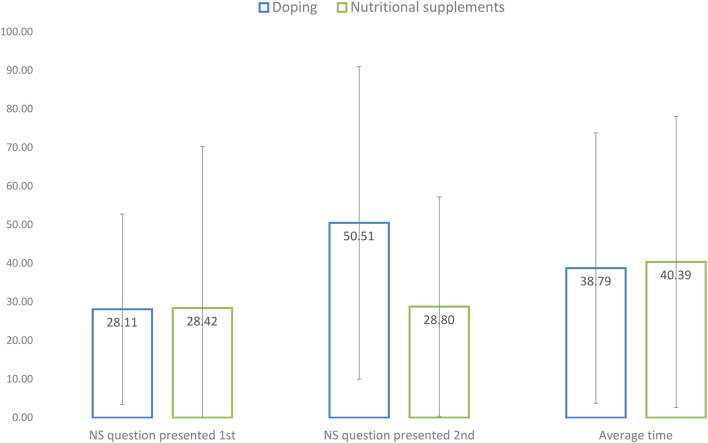
Mean response times for completing the SSC survey model at PAG.

There was no statistically significant difference between the times it took athletes to respond to the doping vs. nutritional supplement question [*F*_(1, 952)_ = 0.004, *p* = 0.948]. However, the order of the question about doping and supplements, as presented at PAG, had a significant interaction effect on the time spent on the doping and nutritional supplement questions [*F*_(1, 952)_ = 324.03, *p* < 0.001]. As the mean response times presented in Figure 2 show, completion time for the doping question was only longer if it was presented first.

Instead of setting an arbitrary cut-off point, we included all completed responses and took fast responding into consideration through analysis. Quick random responding (“clicking through”) is non-compliance with the survey instruction. It is addressed through estimating the proportion of non-compliance and its impact on the estimation figures for doping and the specified supplements rather than omitting them from the analysis. The presence of unrealistically fast responding suggests that not all non-compliance is motivated by deliberate lying about doping use. Therefore, it would be inappropriate to allocate the rate of non-compliers to the estimated prevalence rate of doping.

## Discussion and comparison of prevalence estimations of doping- and nutritional supplement use *via* UQM and SSC indirect estimation models

Prevalence estimates at both WCA and PAG, for both prohibited substances and/or methods and specified supplements estimated from the SSC-derived dataset differ considerably from those reported previously (1). Based on the survey data alone, we cannot be reasonably confident about which estimate is more accurate. We caution that neither should be interpreted as the true prevalence of doping at these sport events because the aim of the study was to develop and test and receive feedback on a survey method from the athletes. Following from this aim, the participation was voluntary and representation of sports or countries in the sample was not a sampling criterion.

Segal's law states that “a man with a watch knows what time it is; a man with two watches is never sure.” Of course, this man with one watch cannot be certain of the time, only he has no way of knowing if he is wrong or not. From a scientific perspective, Segal's law denotes the flaw in dual modular redundancy where critical components of the system are duplicated. Dual modular redundancy caters for the eventuality if one measurement or equipment fails to work. However, when taking two measurements is possible, and the two measurements show different values, then there is no way of knowing which one is correct without a third element—whether it is a third measurement or an external criterion. Hence, in cases where accuracy is critical such as the case of doping prevalence estimation, the triple (or more) modular redundancy rule should be applied. A prevalence estimation for doping with a new assessment tool or method, offering results from a single (new) measurement is not scientifically robust until the assessment tool is validated for its accuracy.

The results from the two studies highlighted the potential weaknesses of both models and helped to enhance our knowledge and understanding of indirect estimation models, their use for prevalence estimate as well as to make further improvements to both models [e.g., (40, 53)]. The UQM has advantage over the SSC by yielding more narrow CIs if honest/correct responding can be assumed. It is however limited in dealing with non-compliance owing to the model being undefined. Non-compliance detection for the UQM model is possible but it requires empirical manipulation with a split sample and two parallel surveys. On the other hand, the SSC can address non-compliance without experimental manipulation, but it is less precise than the UQM and it requires significant post-collection data processing. For the latter, a software for maximum likelihood estimation can be developed to ease the computational demand on potential users.

Because indirect estimation models rely on expected distributions of the non-sensitive information (e.g., birthdays or random numbers), respondents' understanding and compliance with survey instructions, as well as motivation for honest responding is vital yet often overlooked. Retrospectively, we cannot be sure about what happened with these surveys in 2011 or prove how athletes behaved when completed the survey—except that the data contains evidence that non-compliance was present, and was present to a large degree in one of the surveys (WCA). However, we can ask the questions that should be considered in future empirical studies. To raise awareness for the behavioural aspect in indirect estimation surveys, we use the two surveys at hand to open a discussion on the dynamics between deliberate self-protective false responding and the survey format that could explain or at least influence the prevalence estimates.

### Estimated doping and nutritional supplement prevalence at WCA and PAG

To triangulate the estimated prevalence rates, we looked at alternative evidence sources. Available in the public domain, and providing the closest comparison, is prevalence estimation from single-point ABP data taken at the WCA in Daegu with the same population and 2 years later at the WCA in Moscow (59). Based on ABP data, prevalence of blood doping at the two WCAs were estimated as 18% (95% CI: 14–22%) and 15% (95% CI: 12–19%) in 569 endurance athletes in Daegu 2011 and 653 endurance athletes in Moscow 2013, respectively. The prevalence rate for blood doping among endurance athletes at WCA 2011 in Daegu aligns well with the rate estimated by the SSC model for all substance and all athletes at the same event. Whilst the range of substances covered by the survey question is wider (blood doping vs. all prohibited substances and/or methods including blood doping), so is the population (endurance athletes vs. all athletes including the endurance athletes).

**Figure 3:**
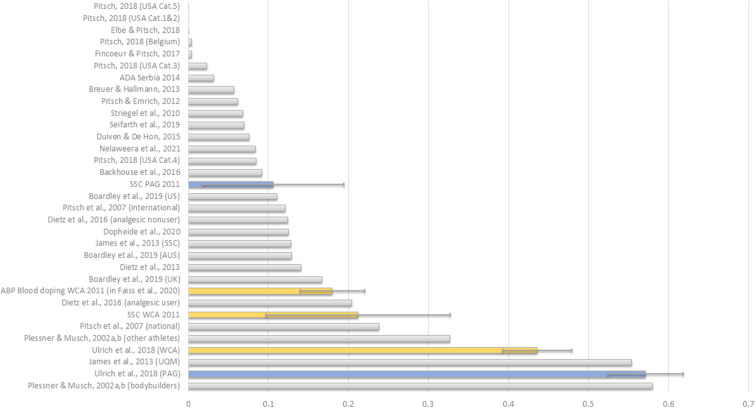
Comparison of doping prevalence estimates for WCA and PAG between the two survey formats, and with literature evidence. Data extracted from Table 1 using the most relevant figure (i.e., elite level and current/last season/past 12 months) when multiple estimates are reported.

Placing the two prevalence estimates in the most relevant literature context, the lower prevalence estimate obtained *via* the SSC appears to be more in line with previous studies—notwithstanding the considerable difference in sports, time, competitive level and population (Figure 3). Figure 3 also illustrates the difference in precision between the SSC and UQM models. The considerably wider confidence intervals for SSC are the direct effect of the model design (i.e., embedding the sensitive question into four innocuous questions). Reducing the number of innocuous questions narrows the confidence intervals but also reduces the level of protection.

Additional sources of evidence may include laboratory reports for athletics for 2010–2011; and for 2011 for countries that participated at the Pan Arab Games in December 2011. Anti-doping rule violation reports for the same sample for samples collected in 2010 and 2011 can also offer a useful reference value. However, the two samples can only partially overlap. Doping testing results from the two events could offer prevalence rates for adverse- and atypical analytical findings for the same population but to our best knowledge, these are not publicly available and may no longer be available due to data protection. Moreover, testing results are limited in assessing true prevalence (3, 4, 60). If such data were available, the likely percentage would be in the 1–3% range.

The admitted use of supplements seems low compared to the literature. One possible explanation for this is that athletes misunderstood the question. Another plausible explanation is that the question specified herbal, mineral, and/or vitamin supplements, thus did not cover all nutritional supplements. We also checked the subsample of athletes who were selected for doping control for comparison. These athletes reported, as part of the doping testing protocol, any use of prescribed-, over the counter medication and dietary supplements in the 3 days prior to providing a sample specimen. Of the 653 logs, ~37% reported using dietary supplements, vitamins or herbal preparations. Given the difference in the time periods (7 days vs. 12 months), it is reasonable to assume that the 12-month use of these substances is higher. It is also reasonable to assume that elite athletes entering a competition where they are more likely to be tested, they stop using supplementation 5 days before the competition to avoid potential positive doping test because of a potential contamination.

### Potential confounding factors

Confounding factors in any empirical investigation can have a marked impact on the measured outcome. Prevalence estimations are not exempt from this. In fact, estimation models appear to be more sensitive than the simpler, direct question formats. Confounding effects could, in principle, arise from the survey format, respondent's behaviour, or both. Survey features such as statement wording, the order in which statements are presented, and marking of listed statements (i.e., numbering) may affect individual responses (61, 62).

The assumptions about the randomisation method or the unrelated innocuous question such as the distribution of the information (e.g., birthdays) is fundamental. In case birthdays are used for randomizations and/or as innocuous question, birthday distribution affect both the SSC and the UQM models, albeit not equally. Limited to international data collection, such as at major sport events, is that in parts of the world (e.g., African countries, parts of Asia), a large proportion of births are not recorded and even more have no birth certificate (63). In the absence of an exact date, often a random date, or January 1st is used on forms [e.g., (64)], which skews the distribution to an unknown degree.

Furthermore, the assumption about what percentage of the respondents answered the sensitive question vs. the unrelated innocuous question is also fundamental, and the UQM is more affected by deliberate (self-protective) non-compliance in this regard than the SSC model because one SSC hypothesis caters for the possibility that respondents only answer the set of innocuous questions. However, in the UQM, as it is illustrated by the study of James et al. (26), when a larger than expected proportion answers the unrelated question, the estimated prevalence will move towards the prevalence for the unrelated question (e.g., birthdays) than the prevalence for the question of interest (the sensitive issue). This might be the culprit of the high, close to 50% prevalence estimation in the 2011 studies at WCA and PAG, with unrecorded or imprecisely recorded birthdays for certain countries and regions. In a scenario where these birthdays were reported as January 1st, then it impacted the UQM model using in WCA and PAG in two ways: first, even with full compliance, larger than the instructed 1/3 of the respondents answered the unrelated question because they were instructed to do so if the birthday of the person they thought of was between the 1st and 10th of the month, then more than 50% of the respondents answered affirmatively the unrelated birthday question (“*Is the person's date of birth in the first half of the year (January through June inclusive)?*” Each of these alone could, in theory, inflate the prevalence estimate to and over 50% simply because of the skewed birthday distribution. The SSC was less affected by this because there was no randomisation, and in the set of four innocuous items, distribution of two items were skewed positively (i.e., birthday in the 1st half of the year, 1st half of the month, and odd number days) and two could have been skewed negatively (i.e., birthday in the 2nd half of the year, 2nd half of the month and even number days).

The survey, just like any other form of self-report, also assumes that respondents possess a precise knowledge of the behaviour they are asked to report on. The challenge is not if athletes remember what they have taken in the previous 12 months, but whether they identified what they used correctly as a prohibited or non-prohibited substance and/or method.

The way the question is asked can also have impact on admission (61, 65, 66). In the present survey (in both the SSC and UQM format), the statement about doping use was carefully formulated to: (1) make it clear that use means intentional use; (2) capture all prohibited substances and methods for a defined period; (3) being neutral by stating the fact (i.e., violated anti-doping rules by using prohibited substances and/or methods) as opposed to making judgements (i.e., “cheated by using prohibited substances and/or methods”); and as such (4) excluded the use of prohibited substances and/or methods in authorised settings (for example out-of-competition if permitted or with the backing of an official Therapeutic Use Exemption). The doping prevalence question assumed that athletes know if they used prohibited substances and/or methods, which is a reasonable assumption given the level of competition at both events. Because the wording was identical in both models, and at WCA the same athletes completed both UQM and SSC versions, any potential lack of knowledge may impact the prevalence estimations but cannot explain the observed difference between the two estimations.

Hypothetically, the order in which the sensitive question is presented, or in the SSC whether statements are numbered (1–5), can also influence the outcome. The position of the sensitive question in the SSC was randomly allocated to prevent inadvertent association between the number of “yes” answers and the position of the sensitive question. It can be further improved by eliminating question numbering. In the UQM model, the innocuous birthday question always preceded the sensitive target (doping), or in PAG, the less sensitive target (dietary supplement), questions. This may have resulted in more athletes answering Question A than expected from the instructions, thus inflating the number of “yes” answers and prevalence estimates (see 30 for a detailed analysis of such scenario).

Furthermore, the complexity of the survey and its instructions require a good reading level which could be even more challenging if athletes complete the survey in a language they are not fluent in. In a highly diverse international environment this challenge is inevitable which simplified language and pictorial support can help. This was not implemented in Daegu or Doha.

### Non-compliance

The SSC model provided clear evidence that non-compliance was present in the WCA study, and detectable—to a lesser degree—in the PAG data. It is reasonable to assume that if a proportion of athletes were non-compliant in the SSC, they were likely to be non-compliant in the UQM as well. An extensive literature exists suggesting that non-compliance occurs to a substantial degree and is indeed an increasingly recognised weakness of the indirect questioning techniques (67). The randomised models, in fact, have been criticised for their susceptibility to non-compliance and efforts have been made to address this shortcoming, along with efforts to improve efficiency [e.g., (21, 22, 39, 54, 68–74)]. It has been shown that models with random noise as extra protection help to alleviate socially desirable responding distortion to a degree, and are hence recommended for situations in which distortion can be expected. Doping, given the social stigma and serious personal consequences, is such an area. Therefore, addressing non-compliance is pivotal to establishing the credibility of the estimates.

The choice of the randomisation device or method is critical to ensuring a high level of compliance. The randomisation must ensure the respondents' trust, but it should also be feasible, accessible, and resistant to manipulation (72). The UQM method with any random person's birthday as randomisation makes self-administration survey application possible and presumably has good level of confidence for protection. However, it is open to manipulation even without lying on the critical question and the true variation in birthdays in the sample at hand should be known, and not merely calculated based on an assumed equal distribution over all days. On the contrary, the SSC contains 4 innocuous but personal questions which afford more flexibility in creating a combination of personal information that is feasible, accessible and ensures the desired level of confidence in respondents (26). In general, respondents are more willing to comply when the model is symmetric (71) as it provides a greater degree of protection when they trust (70, 73), and less likely to respond honestly when the perceived certainty and severity of the sanctions was high (73).

Although the UQM is not a new method, the modification made to the model by allowing respondents to take full control over the randomisation question has not been used, to our best knowledge, except in a study with UK club level athletes (26) where a similarly high, potentially inflated proportion of “yes” answers was observed. This modification empowers respondents to avoid the sensitive question without lying. James et al. (26) provide theoretical evidence that where more than 1/3 of the respondents answer the innocuous unrelated birthday question, whether it is the result of strategic responding or a simple cop-out, there can be a paradox situation where the proportion of “yes” answers to the sensitive question in the UQM is elevated thus giving an inflated estimate when the true prevalence is below 50%. This may be one of the reasons why the estimations for WCA (Daegu) approaches 50%, however it cannot explain the above 50% prevalence estimate for PAG (Doha).

### Efficiency vs. protection of privacy

Through a series of simulations of different indirect estimation models, Ulrich et al. (51) conclude that statistical power is necessary, but not the only condition in indirect estimation models. In survey methodology, there is an inherent trade-off between efficiency and privacy. A model with high level of protection requires sufficient random noise to mask a respondent's response which in turn results in loss of precision (i.e., wider confidence intervals), increased complexity and larger cognitive demand on the respondents. Face validity and mental acceptance of the survey are also important.

The UQM and SSC models illustrate this point well. Although a randomised response model, with its built-in “noise” (i.e., respondents do not have to directly reveal their position on the targeted sensitive behaviour) can offer a buffer against social desirability, some models provide better protection than others. Contrary to the UQM, SSC does not require an answer to the sensitive question as it is embedded among four other potentially affirmative answers. The price to pay for this added protection is some loss in precision. In SSC, protection increases with the number of innocuous items around the single sensitive item, but so is the 95% confidence interval leading to loss in efficiency. The SSC model estimate has a much larger confidence interval than the UQM, yielding a less precise estimate. Consequently, the SSC's five questions mean a larger cognitive load on respondents making it more difficult to keep the information “in their heads.” The trade-off for loss in efficiency is the ability to detect and correct for non-compliance and perhaps greater perceived security.

Furthermore, a more complex and thus more protective survey environment inevitably places greater cognitive demand on respondents and thus increases the response time as well as the risk for non-compliance. However, every participant answers the same set of questions and by answering the sensitive question, the survey has meaning and relevance to the participants. Athletes may not feel comfortable answering questions about their medication and performance enhancement, but the survey “makes sense.” In contrast, UQM is more efficient than the SSC format, yielding more narrow confidence intervals. Protection can be enhanced by increasing the proportion of respondents answering the innocuous unrelated question, but this leads to an increased number of people answering a question (e.g., mother's birthday) that seems unimportant and downright silly. On the one hand, people's willingness to disclose socially sensitive issues or transgressive behaviour is inversely related to the sensitivity of the target question, where sensitivity is simultaneously determined by the intrusiveness, associated risks, and social desirability of the question (75, 76). On the other hand, respondents feel more motivated to take the survey seriously and answer honestly if they feel that their answers are important (77).

### Multiple question sets

Perceived protection in multiple question sets is also linked to the independence of the innocuous questions. In the current study, with only two questions (doping and supplements), meeting this condition was relatively easy with birthday questions and could have been done with one person (i.e., mother's birthday). In case of a larger set (i.e., five questions), the model set-up is more challenging and inevitably needs multiple birthdays (e.g., mother, father, and best friend) or other known randomised parameters.

For example, a multi-question SSC 4+1 model needs sets of 4 independent birthday questions. Let us take one person (e.g., mother), five birthday questions (A–E) with two variants (1 or 2) with 50/50 probability: (A) birthday year is odd or even, (B) month is odd or even, (C) day is odd or even, (D) month is 1–6 or 7–12 (first or second half of the year), and (E) day is 1–15 or 16 and higher (first half of the month or the second). Naturally, the two variants of the same question cannot be used in the same set, or in any set in the same survey because the sets would not be independent. Therefore, if *k* is the number of sensitive questions which are embedded in a unique set of four innocuous question, the number of innocuous birthday questions needed is k^*^4, for which we need to ask about k^*^4/5 birthdays. For five sensitive questions, 20 birthday questions are needed for which we need four individual birthdays. This could be both challenging and overly complex.

### Limitations and recommendations for future research directions

The increased security afforded by the technique only addresses one aspect of the question, which is the reduction in evasive responding but it may not automatically increase the willingness to answer (68, 73, 78). In the current anti-doping climate, high performing athletes who may use prohibited substances and/or methods are not likely motivated to answer, let alone to reveal the truth, about their prohibited behaviour. Simply, they have nothing to gain but much to lose by being honest. Research is needed to explore the conditions which encourage participation and honest responding. Future development could also include comparison of the selected model to other random, non-random and direct methods for (statistical) efficiency. As recommended (79), in cases of comparable privacy protection and efficiency, the selection can be made based on other factors such as complexity, time, cognitive demand on respondents and costs.

Non-compliance is an issue in indirect estimation models, perhaps more so than in direct questioning. Dealing with non-compliance is an interesting computational challenge but its practical relevance is limited. Although non-compliance detection is possible and advisable, correcting for non-compliance is neither simple nor straightforward. The latter requires assumptions about who is non-compliant (i.e., doping users vs. genuine non-users) and how it happens, and the combination of both. Depending on the estimation model, numerous permutations exist and although how well the empirical data fits with each hypothesised scenario for non-compliance can be tested using maximum likelihood (40) or expectation maximisation (58) methods, it is difficult to ascertain that the best fitting model is the true model. It is also possible that more than one scenario fits the data equally well, in which case we have no way of ascertaining which is the one that not only explains the observed distribution but also reflects the behavioural pattern that generated the data at hand. To assist practical application, efforts should be made to refine survey tools built on indirect estimation models to simultaneously: (1) eliminate or at least minimise non-compliance with survey instructions for “innocent” reasons, and (2) make motivated non-compliance (self-protective lying) effortful. The former criterion favours models that demand low cognitive effort (e.g., simple format and uncomplicated instructions), whereas the latter favours models where self-protective “no” saying strategy is not obvious.

The validity of the data obtained *via* indirect estimation surveys can also be improved with a practice question that precedes the question(s) of interest. Carefully crafted, this question can also be used to detect the degree by which respondents do not follow the survey instructions. For example, the question can be set to 100% “yes” or 100% “no” answer to a non-sensitive attribute (e.g., asking “Are you a competitive athlete?” for a definite “yes” or “Are you retired from sport?” for a definite “no” when data are collected at a major sport event among competitors). The importance of a comprehension cheque is underscored by the results from an experimental study comparing brief vs. comprehensive instructions in one indirect estimation model (crosswise model) and direct questioning (80). The results showed that both false negatives and false positives were present. Congruently with the literature, false negative responding (denial of a sensitive behaviour) was present in all question formats but to a lesser degree in the indirect format than in direct questioning. False positive responses (incorrectly saying “yes”) appear to be linked to understanding and can be reduced with detailed instructions and comprehension cheques among the highly educated. Although this study specifically focused on one indirect questioning format, the crosswise model, it is likely the other indirect estimation models would fit to the same pattern to a varying degree. Further research is advisable to elucidate the degree by which different indirect estimation models are affected. In addition, Petróczi and Haugen (81) previously argued that “lying” in self-reported surveys is not exclusive to the “guilty” and denial. Respondents may falsely admit doping use if it is safe to do so to distort the result, thus response bias simultaneously can lead to both under- and over-reporting. Since the potential impact of deliberate (but false admission of doping cannot be ruled out, it also deserves attention in investigating potential bias. For practical applications, models that are less prone to false positives owing to poor comprehension as well as false negatives owing to lack of comprehension (non-motivated false reporting) and fear of exposure (motivated false reporting) are more advantageous.

Future empirical studies are required to elucidate how athletes manipulate their responses on the SSC survey if they have a sensitive or discriminating behaviour to hide, as opposed to simply not following the instructions. Having a smaller sample with known doping prevalence or other substance abuse *via* biomarkers such as hair, saliva, exhaled breath or dried blood spots. For example, hair samples from 500 athletes with carefully matched target question for both substance (e.g., anabolic steroids) and timeframe taking hair growth into consideration could be used for model testing and validation (8, 82–84). Whilst the target question must be matched to the bioanalysis in terms of substance and timeframe, it is not required that the survey answers and bioanalysis results are matched individually because SSC responses are not useful at the individual level, which may facilitate the recruitment of volunteers. However, the sample with SSC results and the sample with bioanalysis results must overlap perfectly. It must be noted if some bioanalysis is used for validation, the performance of the prediction model is compared to the bioanalysis, not to the “absolute truth” about doping prevalence—but this is likely to be the best available option for validation. Therefore, the closer the bioanalysis can be to the absolute truth prevalence in the selected sample, the better it is for validation.

An alternative approach is to survey known doping users where the doping prevalence is expected to be 100%. This approach has been used, for example, with known abusers of the welfare and unemployment benefit system (85). Although the large proportion of the known offenders denied benefit fraud despite the obvious, this method is better suited to evidence weakness of the survey method than it could be for showing accuracy. First, in order to simulate a real survey situation, respondents must believe that they are not targeted specifically because of their previous positive doping test but received the survey as one of the elite athlete sampling pool. Even if this is achieved, the sample is biased towards those who have no reason to lie about their doping behaviour apart from social desirability as they are already known to having used doping at one point in their life.

## General discussion

The widely different estimates for doping present a situation where—despite having a much better understanding and much improved tools—uncertainties still exist about doping prevalence at these two events. Based on the available data, it is not possible to identify which estimate (if any) is closer to the true prevalence. Additional studies are required to investigate the nature of non-compliance and cheating. An additional study, even if on a smaller scale where absolute objective verification *via* some biomarkers is available, is needed to answer the “which one of the models gives a better estimate” question with at least reasonable certainty.

The large observed discrepancy between the prevalence estimations for the same population, coupled with sampling procedure warrant caution in interpreting these estimates as *bona fide* prevalence rates. To make sense of this peculiar situation, we speculate that the divergence between the results is probably and inadvertently affected by allowing athletes to take control over an element of the randomisation in the UQM but not the SSC, which in turn has highlighted the need for detailed attention to the survey format. Altogether, the results suggest that higher estimated prevalence rates do not guarantee that they are more reliable or valid than lower rates from different approaches (55, 86).

We acknowledge that we are in a privileged position with valuable insight. Our intention is not to criticise the former, nor to promote the alternative method. Rather, we intend to facilitate further research and to promote a holistic approach in future empirical endeavours with the view to improve into the validity and reliability of prevalence surveys relying on an indirect estimation model. Through sketching hypothetical scenarios and contesting fundamental assumptions, we drew attention to the importance of the potential confounding effect of structural factors, the “human side” and the interplay between the two to ensure that future attempts of estimating doping prevalence are less affected by these confounding factors. Although confounding effects cannot be eliminated, they can (and should) be controlled for and reduced as much as possible.

Although non-compliance can be modelled and taken into account, such analyses inevitably rely on a host of assumptions and their unknown combinations. As we demonstrated in this paper, adjusting prevalence estimates for non-compliance, although analytically possible, can lead to multiple prevalence rates which are only statistically but not ecologically plausible. Without any independent indicator for how respondents behaved, there is no way to select the most likely scenario. Consequently, non-compliance adjusted estimations only show what prevalence rate is possible to estimate from the empirical data at hand, not what the prevalence rate is. Therefore, the pragmatic goal for future improvement is to reduce non-compliance as much as possible by addressing complexity, language barrier, potential misunderstanding, along with leaving no room for alternative interpretations of the rules. Further research into both cognitive and behaviour aspects is warranted to improve the ecological validity of prevalence rates obtained *via* indirect estimation models.

## Conclusion

Concluding the available evidence from the literature, as well as from the empirical work presented in this article, we must paraphrase Pielke's position (15): reliable and validated method for “*quantifying prevalence and how it changes over time* is possible, but not yet readily available.” In line with emergent challenges to the validity of randomised/fuzzy response model estimates, we recommend critical appraisal of the obtained prevalence rates and triangulation with other sources as a superior approach to the customary “the higher rate must be closer to the truth” heuristics. The accuracy of prevalence estimates of highly sensitive behaviour appears to hinge on human factors such as understanding and compliance with instructions, as well as potential motivation for honesty. We further argue that non-compliance—both motivated and unmotivated—is the Achilles heel of indirect estimation models, which calls for attention before sector-wide application.

## Data availability statement

The data analysed for this study can be found within the article and in Ulrich et al. (1) [https://rdcu.be/cgSXw]: Supplementary material 1 [https://static-content.springer.com/esm/art%3A10.1007%2Fs40279-017-0765-4/MediaObjects/40279_2017_765_MOESM1_ESM.pdf] and Supplementary material 2 [https://static-content.springer.com/esm/art%3A10.1007%2Fs40279-017-0765-4/MediaObjects/40279_2017_765_MOESM2_ESM.xlsx].

## Ethics statement

The studies involving human participants were reviewed and approved by Research Ethics Committee Faculty of Science, Engineering and Computing, Kingston University London. Written informed consent for participation was not required for this study in accordance with the national legislation and the institutional requirements.

## Author contributions

AP conceptualised the method, supervised the data collection and analysis, and drafted the manuscript. MC contributed to data analysis, interpretation of the results, and drafting the manuscript. OH, DS, and MS contributed to interpretation of the results and drafting the manuscript. All authors have critically reviewed and approved the final version of the manuscript.

## Funding

In-kind support from the World Anti-Doping Agency made the software development and data collection possible. Publication costs was covered by Research and Expertise in antiDoping sciences (REDs), Institute of Sport Sciences, University of Lausanne.

## Conflict of interest

AP was a member of the first WADA Working Group on Doping Prevalence between 2011 and 2012 and she chairs the current WADA Working Group on Doping Prevalence since 2017. OH, MS, and DS are members the current WADA Working Group on Doping Prevalence since 2017. MC is an *ad hoc* member of the WADA Working Group on Doping Prevalence. Working Group members receive no salary for their work for WADA but are entitled to expenses covered and to receive indemnity for formal meetings and up to five days per year for preparation.

## Publisher's note

All claims expressed in this article are solely those of the authors and do not necessarily represent those of their affiliated organizations, or those of the publisher, the editors and the reviewers. Any product that may be evaluated in this article, or claim that may be made by its manufacturer, is not guaranteed or endorsed by the publisher.

## Footnotes

^1^AP was also a member of the previous Working Group between 2011 and 2012.

## References

[1] Ulrich R Pope HG Cléret L Petróczi A Nepusz T Schaffer J. Doping in two elite athletics competitions assessed by Randomized-Response surveys. Sports Med. (2018) 48:211–9. 10.1007/s40279-017-0765-4 28849386

[2] World Anti-Doping Code. World Anti-Doping Code Report. (2021). Available online at: https://www.wada-ama.org/sites/default/files/resources/files/2021_wada_code.pdf (accessed August 7, 2022).

[3] De Hon O Kuipers H van Bottenburg M. Prevalence of doping use in elite sports: a review of numbers and methods. Sports Med. (2015) 45:57–69. 10.1007/s40279-014-0247-x 25169441

[4] Gleaves J Petróczi A Folkerts D De Hon O Macedo E Saugy M. Doping prevalence in competitive sport: evidence synthesis with “best practice” recommendations and reporting guidelines from the WADA Working Group on Doping Prevalence. Sports Med. (2021) 21:1477. 10.1007/s40279-021-01477-y 33900578

[5] Chaudhuri A Christofides TC. Indirect techniques as alternatives to randomized response. In:A Chadhuri TC Christofides, editors, *Indirect Questioning in Sample Surveys*. Berlin, Heidelberg: Springer. (2013). p. 115–49. 10.1007/978-3-642-36276-7_6

[6] Lensvelt-Mulders GJ Hox JJ Van der Heijden PG Maas CJ. Meta-analysis of randomized response research: thirty-five years of validation. Sociol Method Res. (2005) 33:319–48. 10.1177/0049124104268664

[7] Greenberg BG Abul-Ela ALA Simmons WR Horvitz DG. The unrelated question randomized response model: theoretical framework. J Am Stat Assoc. (1969) 64:520–39. 10.1080/01621459.1969.10500991

[8] Petróczi A Nepusz T Cross P Taft H Shah S Deshmukh N. New non-randomised model to assess the prevalence of discriminating behaviour: a pilot study on mephedrone. Subst Abuse Treat Prev Policy. (2011) 6:20. 10.1186/1747-597X-6-20 21812979 PMC3163613

[9] Rohan T. Antidoping Agency Delays Publication of Research. New Your Times. (2013). Available online at: https://www.nytimes.com/2013/08/23/sports/research-finds-wide-doping-study-withheld.html (accessed August 22, 2013).

[10] DCMS Committee. Committee Publishes “Blocked” Study on Doping. UK Parliamentary Committee, Digital Culture, Media Sport. (2015). Available online at: https://committees.parliament.uk/committee/378/digital-culture-media-and-sport-committee/news/105044/committee-publishes-blocked-study-on-doping/ (accessed September 8, 2015).

[11] Pielke R Jr Boye E. Scientific integrity and anti-doping regulation. Int J Sport Policy Polit. (2019) 11:295–313. 10.1080/19406940.2019.159696831326919

[12] Pitsch W Gleaves J. If you're not first. You're last: Are the empirical premises correct in the ethics of anti-doping? Sport Ethics Phil. (2021) 15:495–506. 10.1080/17511321.2020.1818277

[13] Read D Skinner J Lock D Houlihan B. Legitimacy driven change at the world anti-doping agency. Int J Sport Policy Politics. (2019) 11:233–45. 10.1080/19406940.2018.1544580

[14] Heuberger JA Henning A Cohen AF Kayser B. Dealing with doping. A plea for better science, governance and education. Br J Clin Pharmacol. (2022) 88:566–78. 10.1111/bcp.14998 34291479

[15] Pielke R. Assessing doping prevalence is possible. So what are we waiting for? Sports Med. (2018) 48:207–9. 10.1007/s40279-017-0792-1 28983882

[16] Warner SL. Randomized response: a survey technique for eliminating evasive answer bias. J Am Stat Assoc. (1965) 60:63–6. 10.1080/01621459.1965.10480775 12261830

[17] Elbe AM Pitsch W. Doping prevalence among Danish elite athletes. Perf Enhanc Health. (2018) 6:28–32. 10.1016/j.peh.2018.01.001

[18] Striegel H Ulrich R Simon P. Randomized response estimates for doping and illicit drug use in elite athletes. Drug Alcohol Depend. (2010) 106:230–2. 10.1016/j.drugalcdep.2009.07.026 19740612

[19] Fincoeur B Pitsch W. Omgaan met sociale wenselijkheid: Inschatting van de dopingprevalentie aan de hand van de Randomized Response Technique. Panopticon. (2017) 38:376–86. Available online at: http://www.maklu-online.eu/en/tijdschrift/panopticon/jaargang-volume-38/issue-5-september-october-2017/omgaan-met-sociale-wenselijkheid-inschatting-van-d/

[20] Nilaweera A Nadishani U Nipunya G Wijekoon N. Knowledge, attitude and usage of doping drugs among national level athletes in Sri Lanka. Br J Sports Med. (2021) 55:A136–A136. 10.1136/bjsports-2021-IOC.327

[21] Pitsch W Emrich E Klein M. Zur Häufigkeit des Dopings im Leistungssport: Ergebnisse eines www-surveys. Leipziger Sportwissenschaftliche Beiträge. (2005) 46:63–77.

[22] Pitsch W Emrich E. The frequency of doping in elite sport: results of a replication study. Int Rev Sociol Sport. (2012) 47:559–80. 10.1177/1012690211413969

[23] Pitsch W Maats P Emrich E. Zur Häufigkeit des Dopings im deutschen Spitzensport – eine Replikationsstudie. In:E Emrich W Pitsch, editors, *(Hrsg.): Sport und Doping. Zur Analyse einer antagonistischen Symbiose*. Frankfurt am Main u. a. (2009). p. 19–36.

[24] Pitsch W Maats P Emrich E. Zur Häufigkeit des Dopings im deutschen Spitzensport. Magazin Forschung. (2009) 1:15–9.

[25] Pitsch W Emrich E Klein M. Doping in elite sports in Germany: results of a www survey. Eur J Sport Soc. (2007) 4:89–102. 10.1080/16138171.2007.11687797

[26] James RA Nepusz T Naughton DP Petróczi A. A potential inflating effect in estimation models: Cautionary evidence from comparing performance enhancing drug and herbal hormonal supplement use estimates. Psychol Sport Exerc. (2013) 14:84–96. 10.1016/j.psychsport.2012.08.003

[27] Boardley ID Smith AL Ntoumanis N Gucciardi DF Harris TS. Perceptions of coach doping confrontation efficacy and athlete susceptibility to intentional and inadvertent doping. Scand J Med Sci Sport. (2019) 29:1647–54. 10.1111/sms.13489 31148275

[28] Dietz P Ulrich R Dalaker R Striegel H Franke AG Lieb K. Associations between physical and cognitive doping—a cross-sectional study in 2997 triathletes. PLoS ONE. (2013) 8:e78702. 10.1371/journal.pone.0078702 24236038 PMC3827233

[29] Dietz P Dalaker R Letzel S Ulrich R Simon P. Analgesics use in competitive triathletes: its relationship to doping and on predicting its usage. J Sports Sci. (2016) 34:1965–9. 10.1080/02640414.2016.1149214 26911564

[30] Seifarth S Dietz P Disch AC Engelhardt M Zwingenberger S. The prevalence of legal performance-enhancing substance use and potential cognitive and or physical doping in German recreational triathletes, assessed *via* the randomised response technique. Sports. (2019) 7:241. 10.3390/sports7120241 31779150 PMC6956052

[31] Backhouse SH Whittaker LS McKenna J Robinson S Begs C Petróczi A. Schoolboy Supplements Use Behaviours Doping Vulnerability. Retrieved from the Rugby Football Union research archives, London. Retrieved from Leeds Beckett University. (2016). Available online at: https://eprints.leedsbeckett.ac.uk/id/eprint/7554/1/SchoolboySupplementUseBehavioursAndDopingVulnerabilityPV-BACKHOUSE.pdf (accessed July 17, 2021).

[32] Antidoping Agency of Serbia. Who is your team? The Importance of “Sport Entourage” for Sport Fellows of Serbia. Recommendations to Ministry of Youth and Sports. Ministry of Youth and Sports: Belgrade. (2014).

[33] Dopheide M Elling A Balk L. Antidopingbeleid in de Nederlandse topsport. Ervaringen en percepties van (oud-)topsporters. Mulier Instituut. (2020). Available online at: https://www.kennisbanksportenbewegen.nl/?file=9987&m=1587648009&action=file.download (accessed July 17, 2020).

[34] Duiven E De Hon O. De Nederlandse topsporter en het anti-dopingbeleid 2014 – 2015. Dopingautoriteit. (2015). Available online at: https://www.dopingautoriteit.nl/media/files/2015/Topsportonderzoek_doping_2015-07-21_DEF.pdf (accessed July 17, 2021).

[35] Breuer C Hallmann K. Dysfunktionen des spitzensports: doping, match-fixing und gesundheitsgefährdungen aus sicht von bevölkerung und athleten. Bundesinst. für Sportwissenschaft. (2013).

[36] Plessner H Musch J. Wie verbreitet ist Doping im Leistungssport? Eine www Umfrage mit Hilfe der Randomized-Response-Technik. In:B Strauß, editors, *Expertise im Sport*. Cologne: bps (2002). p. 78–9.

[37] Plessner H Musch J. Randomized Response Investigation of the Prevalence of Doping. (2002).17034444

[38] Boruch RF. Assuring confidentiality of responses in social research: a note on strategies. Am Sociol. (1971) 6:308–11.

[39] Clark SJ Desharnais RA. Honest answers to embarrassing questions: detecting cheating in the randomized response model. Psychol Method. (1998) 3:160–8. 10.1037/1082-989X.3.2.160

[40] Nepusz T Petróczi A Naughton DP Epton T Norman P. Estimating the prevalence of socially sensitive behaviors: attributing guilty and innocent noncompliance with the single sample count method. Psychol Methods. (2014) 19:334–55. 10.1037/a0034961 24295152

[41] Kuk AYC. Asking sensitive questions indirectly. Biomerika. (1990) 77:436–8. 10.1093/biomet/77.2.436

[42] Wolff W Sandouqa Y Brand R. Using the simple sample count to estimate the frequency of prescription drug neuroenhancement in a sample of Jordan employees. Int J Drug Pol. (2016) 31:51–5. 10.1016/j.drugpo.2015.12.014 26818080

[43] Zamanian M Zolala F Haghdoost AA Baneshi MR. Estimating the annual abortion rate in kerman, Iran: comparison of direct, network scale-up, and single sample count methods. Int J Fertil Steril. (2019) 13:209–14. 10.22074/ijfs.2019.5721 31310075 PMC6642432

[44] Epton T Norman P Dadzie A-S Harris PR Webb TL Sheeran P. A theory-based online health behaviour intervention for new university students (U@Uni): results from a randomised controlled trial. BMC Pub Health. (2014) 14:563. 10.1186/1471-2458-14-563 24903620 PMC4067627

[45] Cameron D Epton T Norman P Sheeran P Harris PR Webb TL. A theory-based online health behaviour intervention for new university students (U@Uni: LifeGuide): results from a repeat randomized controlled trial. Trials. (2015) 16:555. 10.1186/s13063-015-1092-4 26643917 PMC4672536

[46] Cross P St John FAV Khan S Petroczi A. Innovative techniques for estimating illegal activities in a human-wildlife-management conflict. PLoS ONE. (2013) 8:e53681. 10.1371/journal.pone.0053681 23341973 PMC3547042

[47] Ibbett H Jones JP St John FA. Asking sensitive questions in conservation using Randomised Response Techniques. Biol Conserv. (2021) 260:109191. 10.1016/j.biocon.2021.109191 34404956 PMC8346952

[48] Lensvelt-Mulders GJ Hox JJ Van Der Heijden PG. How to improve the efficiency of randomised response designs. Qual Quant. (2005) 39:253–65. 10.1007/s11135-004-0432-3

[49] Oral E. Surveying sensitive topics with indirect questioning. In:JP Hessling, editor, *Statistical Methodologies*. London: IntechOpen. (2019) 1–28. 10.5772/intechopen.8452432603352

[50] Pitsch W. Minimizing response bias: an application of the randomized response technique. In:V Barkoukis L Lazuras H Tsorbatzoudis, editors, *The Psychology of Doping in Sport*. London: Routledge. (2015). p. 137–52.

[51] Ulrich R Schröter H Striegel H Simon P. Asking sensitive questions: a statistical power analysis of randomized response models. Psychol Methods. (2012) 17:623–41. 10.1037/a0029314 22924599

[52] Nuno A St John FA. How to ask sensitive questions in conservation: a review of specialized questioning techniques. Biol Conserv. (2015) 189:5–15. 10.1016/j.biocon.2014.09.047

[53] Reiber F Schnuerch M Ulrich R. Improving the efficiency of surveys with randomized response models: a sequential approach based on curtailed sampling. Psychol Methods. (2020) 2020:353. 10.1037/met0000353 32915000

[54] Pitsch W. Assessing and explaining the doping prevalence in cycling. In:B Fincoeur J Gleaves F Ohl, editors, *Doping in Cycling: Interdisciplinary Perspectives*. London: Routledge. (2018). p. 13–30. 10.4324/9781351103879-2

[55] Höglinger M Jann B. More is not always better: an experimental individual-level validation of the randomized response technique and the crosswise model. PLoS ONE. (2018) 13:e0201770. 10.1371/journal.pone.0201770 30106973 PMC6091935

[56] Sagoe D Cruyff M Spendiff O Chegeni R de Hon O Saugy M. Functionality of the Crosswise Model for assessing sensitive or transgressive behavior: a systematic review and meta-analysis. Front Psychol. (2021) 12:655592. 10.3389/fpsyg.2021.655592 34248750 PMC8260852

[57] Bernard HR Hallett T Iovita A Johnsen EC Lyerla R McCarty C. Counting hard-to-count populations: the network scale-up method for public health. Sex Transm Infect. (2010) 86:11–5. 10.1136/sti.2010.044446 21106509 PMC3010902

[58] Groenitz H. Improvements and extensions of the item count technique. Electron J Stat. (2014) 8:2321–51. 10.1214/14-EJS951

[59] Faiss R Saugy J Zollinger A Robinson N Schuetz F Saugy M. Prevalence estimate of blood doping in elite track and field athletes during two major international events. Front Physiol. (2020) 11:160. 10.3389/fphys.2020.00160 32161553 PMC7052379

[60] Petróczi A Gleaves J De Hon O Sagoe D Saugy M. Prevalence of doping in sport. In:D Mottram N Chester, editors, *Drugs in Sport*. 8th ed. London: Routledge. (2021). p. 4. 10.4324/9781003096160-4

[61] Guo Y Kopec JA Cibere J Li LC Goldsmith CH. Population survey features and response rates: a randomized experiment. Am J Public Health. (2016) 106:1422–6. 10.2105/AJPH.2016.303198 27196650 PMC4940641

[62] Peytchev A. Survey breakoff. Public Opin Q. (2009) 73:74–97. 10.1093/poq/nfp014

[63] UNICEF. Birth Registration for Every Child by 2030: Are We on Track? (2020). Available online at: https://www.unicef.org/media/62981/file/Birth-registration-for-every-child-by-2030.pdf (accessed August 7, 2022).

[64] Sieff K. In Afghanistan, Jan. 1 Is Everyone's Birthday. Washington Post. (2013). Available online at: https://www.washingtonpost.com/world/in-afghanistan-its-everyones-birthday/2013/12/31/81c18700-7224-11e3-bc6b-712d770c3715_story.html (accessed December 31, 2013).

[65] Kanayama G Boynes M Hudson JI Field AE Pope HG Jr. Anabolic steroid abuse among teenage girls: an illusory problem? Drug Alcohol Depend. (2007) 88:156–62. 10.1016/j.drugalcdep.2006.10.013 17127018 PMC1978191

[66] Petróczi A. The doping mindset–Part II: potentials and pitfalls in capturing athletes' doping attitudes with response-time methodology. Perform Enhanc Health. (2013) 2:164–81. 10.1016/j.peh.2014.08.003

[67] Krause T Wahl A. Non-compliance with indirect questioning techniques. Survey Res Methods. (2022) 16:45–60. 10.18148/srm/2022.v16i1.782434404956

[68] Böckenholt U Barlas S Van Der Heijden PG. Do randomized-response designs eliminate response biases? An empirical study of non-compliance behavior. J Appl Econ. (2009) 24:377–92. 10.1002/jae.1052

[69] Cruyff MJ van den Hout A van der Heijden PG Böckenholt U. Log-linear randomized-response models taking self-protective response behavior into account. Sociol Method Res. (2007) 36:266–82. 10.1177/0049124107301944

[70] Landsheer JA Van Der Heijden P Van Gils G. Trust and understanding, two psychological aspects of randomized response. Qual Quant. (1999) 33:1–12. 10.1023/A:100436181997436007340

[71] Ostapczuk M Moshagen M Zhao Z Musch J. Assessing sensitive attributes using the randomized response technique: evidence for the importance of response symmetry. J Educ Behav Stat. (2009) 34:267–87. 10.3102/1076998609332747

[72] Moshagen M Hilbig BE Musch J. Defection in the dark? A randomized-response investigation of cooperativeness in social dilemma games. Eur J Soc Psychol. (2011) 41:638–44. 10.1002/ejsp.793

[73] Böckenholt U Van der Heijden PG. Item randomized-response models for measuring noncompliance: risk-return perceptions, social influences, and self-protective responses. Psychometrika. (2007) 72:245–62. 10.1007/s11336-005-1495-y

[74] Ostapczuk M Musch J Moshagen M. Improving self-report measures of medication non-adherence using a cheating detection extension of the randomised-response-technique. Stat Methods Med Res. (2011) 20:489–503. 10.1177/0962280210372843 20639269

[75] Tourangeau R Rips LJ Rasinski K. The Psychology of Survey Response. Cambridge: Cambridge University Press. (2000). 10.1017/CBO9780511819322

[76] Tourangeau R Yan T. Sensitive questions in surveys. Psychol Bull. (2007) 133:859–83. 10.1037/0033-2909.133.5.859 17723033

[77] Brueckner S Petróczi A. Faulty assumptions and the potential inflating effect on doping prevalence figures: exploring noncompliance in the Unrelated Question Model. In: Jahrestagung der Arbeitsgemeinschaft für Sportpsychologie (ASP) Conference, May 25 – 27, 2017. Bern (2017).

[78] Peeters CF Lensvelt-Mulders GJ Lasthuizen K. A note on a simple and practical randomized response framework for eliciting sensitive dichotomous and quantitative information. Sociol Methods Res. (2010) 39:283–96. 10.1177/00491241103780

[79] Guerriero M Sandri MF. A note on the comparison of some randomized response procedures. J Stat Planning Infer. (2007) 137:2184–90. 10.1016/j.jspi.2006.07.004

[80] Meisters J Hoffmann A Musch J. Can detailed instructions and comprehension checks increase the validity of crosswise model estimates? PLoS ONE. (2020) 15:e0235403. 10.1371/journal.pone.0235403 32603352 PMC7326177

[81] Petróczi A Haugen KK. The doping self-reporting game: the paradox of a ‘false-telling' mechanism and its potential research and policy implications. Sport Manage Rev. (2012) 15:513–7. 10.1016/j.smr.2012.04.002

[82] Kintz P Gheddar L Ameline A Arbouche N Raul JS. Hair testing for doping agents. What is known and what remains to do. Drug Test Anal. (2020) 12:316–22. 10.1002/dta.2766 31943812

[83] Shah I Petroczi A Uvacsek M Ránky M Naughton DP. Hair-based rapid analyses for multiple drugs in forensics and doping: application of dynamic multiple reaction monitoring with LC-MS/MS. Chem Cent J. (2014) 8:73. 10.1186/s13065-014-0073-0 25530799 PMC4272537

[84] Vargo EJ Petróczi A Shah I Naughton DP. It is not just memory: propositional thinking influences performance on the autobiographical IAT. Drug Alcohol Depend. (2014) 145:150–5. 10.1016/j.drugalcdep.2014.10.008 25457738

[85] Van der Heijden PG Van Gils G Bouts J Hox JJ. A comparison of randomized response, computer-assisted self-interview, and face-to-face direct questioning: Eliciting sensitive information in the context of welfare and unemployment benefit. Sociol Methods Res. (2000) 28:505–37. 10.1177/004912410002800400

[86] Höglinger M Diekmann A. Uncovering a blind spot in sensitive question research: false positives undermine the crosswise-model RRT. Pol Anal. (2017) 25:131–7. 10.1017/pan.2016.5

