# Corrigendum: Hidden figures: Revisiting doping prevalence estimates previously reported for two major international sport events in the context of further empirical evidence and the extant literature

**DOI:** 10.3389/fspor.2023.1129320

**Published:** 2023-01-31

**Authors:** Andrea Petróczi, Maarten Cruyff, Olivier de Hon, Dominic Sagoe, Martial Saugy

**Affiliations:** ^1^ School of Life Sciences, Pharmacy and Chemistry, Faculty of Health, Science, Social Care and Education, Kingston University, London, United Kingdom; ^2^ Department of Movement Sciences, Faculty of Movement and Rehabilitation Sciences, Katholieke Universiteit (KU) Leuven, Leuven, Belgium; ^3^ Willibald Gebhardt Research Institute, University of Münster, Münster, Germany; ^4^ Faculty of Social Sciences, Utrecht University, Utrecht, Netherlands; ^5^ Doping Authority Netherlands, Capelle aan den IJssel, Netherlands; ^6^ Department of Psychosocial Science, University of Bergen, Bergen, Norway; ^7^ Research and Expertise in anti-Doping Sciences (REDs), Institute of Sport Sciences, University of Lausanne, Lausanne, Switzerland

**Keywords:** athlete, performance enhancement, doping, randomised response technique, prevalence, single sample count, prohibited substance, elite sport

Corrigendum on Hidden figures: Revisiting doping prevalence estimates previously reported for two major international sport events in the context of further empirical evidence and the extant literature By Petróczi A, Cruy M, de Hon O, Sagoe D and Saugy M (2022) Front. Sports Act. Living 4:1017329. doi: 10.3389/fspor.2022.1017329

**Correction to Table/Figure**

In the published article, there was an error in table 4 as published. There was an error in the estimated % of admitted doping (*d*) for H2: 0.3031 ± 0.0800 was erroneously listed for both the estimated % of admitted doping (*d*) and for the estimated % of noncompliance (*nc*). The correct value for the estimated % of admitted doping (*d*) is 0 and the value of the estimated % of noncompliance (*nc*) remains 0.3031 ± 0.0800**.** The corrected Table 4 and its caption Estimated use of prohibited performance enhancing substances and/or methods at WCA (12-month prevalence) appear below.

**Table 4:** Estimated use of prohibited performance enhancing substances and/or methods at WCA (12-month prevalence).

	Model fit			Estimated % of admitted doping (*d*)	Estimated % of noncompliance (*nc*) *p = 1.1E-16*
H	Log-likelihood	AIC	BIC		
H0	−1854.0342	3708.07	3708.07		
H1	−1853.9311	3709.86	3714.95	0.0132 ± 0.0576	0
H2	−1819.0675	3642.14	3652.32	0	0.3031 ± 0.0800
H3	−1819.5104	3643.02	3653.21	0.0132 ± 0.0576	0.0699 ± 0.0201
**H4**	**−1799**.**2650**	**3602**.**53**	**3612**.**72**	**0.2124 **±** 0.0873**	**0.3190 **±** 0.0562**
H5	−1853.9311	3711.86	3722.05	0.0132 ± 0.0576	0

*In Figure 3*: Comparison of doping prevalence estimates for WCA and PAG between the two survey formats, and with literature evidence. Data extracted from Table 1 using the most relevant figure (i.e., elite level and current/last season/past 12 months) when multiple estimates are reported. In the figure legend ‘ABP Blood doping WAC 2021’ should read as ‘ABP Blood doping WCA 2011’.

**Incorrect Supplementary Material**

In the published article, there was an error in Supplementary Material 2: Detailed prevalence and noncompliance estimations, Table 2.1: Detailed prevalence and noncompliance estimations for WAC and PAG, 2011. Incorrect values were reported for ‘Estimation’. The correct material statement appears below.

**Table:** 

Model			WAC doping use	PAG doping use	PAG nutritional supplement use
**Estimation**	***d***	Lowest	0.1251	0.0290	0.0103
	*** ***	Highest	0.2997	0.1836	0.1611
	*** ***	Midpoint of multiplied Cis	0.2124	0.1063	0.0857
	***CI (d)***	0.0872	0.0773	0.0754
	***nc***	Lowest	0.2628	0.0927	0.0531
	*** ***	Highest	0.3752	0.1043	0.1755
	*** ***	Midpoint of multiplied Cis	0.3190	0.0985	0.1143
	***CI (nc)***	0.0562	0.0058	0.0612
**D**	d*nc	Lowest	0.03287628	0.00268714	0.00054693
	*** ***	Highest	0.11244744	0.019156824	0.02827305
	*** ***	Midpoint of multiplied Cis	0.07266186	0.010921982	0.01440999
	d* (1-nc)	Lowest	0.07816248	0.02597414	0.00849235
	*** ***	Highest	0.22093884	0.166587624	0.15254559
	*** ***	Midpoint of multiplied Cis	0.14955066	0.096280882	0.08051897
**NC**	(1-d)*nc	Lowest	0.18403884	0.075647624	0.04454559
	*** ***	Highest	0.32826248	0.101314104	0.17369235
	*** ***	Midpoint of multiplied CIs	0.25615066	0.088480882	0.10911897
	(1-d)* (1-nc)	Lowest	0.43754744	0.731216824	0.69167305
	*** ***	Highest	0.64497628	0.881027140	0.93714693
	*** ***	Midpoint of multiplied CIs	0.54126186	0.806121982	0.81440999

The authors apologize for these error and state that this does not change the scientific conclusions of the article in any way. The original article has been updated.

