# Supplementary material for: Hidden figures: Revisiting doping prevalence estimates previously reported for two major international sport events in the context of further empirical evidence and the extant literature
Source: Front Sports Act Living. 2022 Dec 5;4:1017329. doi: 10.3389/fspor.2022.1017329 (PMC9760848; doi:10.3389/fspor.2022.1017329)
Supplement: Supplementary file 1 [file Data_Sheet_1.pdf]

Supplementary Material 1: The Single Sample Count (SSC) method

The SSC (Petróczi et al., 2011) is a variation of a fuzzy response model known as the Unmatched Count (Dalton et al., 1994). In the Unmatched Count model, the sensitive question or statement is embedded in a certain number of unrelated innocuous questions/statements and respondents are instructed not to answer each question/statement independently but to only indicate the total number of affirmative answers. Participants are allocated to one of two groups where only one group receives the sensitive question. The control group receives the innocuous questions only. As participants are randomly allocated to either the experimental or the control group, the two groups are similar. Prevalence of the sensitive issue is then calculated from the difference in the number of affirmative answers between the two groups. The control group serves no other purpose than the baseline distribution for the innocuous questions resulting in considerable 'waste' of data and potential resentment from participants as half the sample only responds to unrelated questions.

The SSC addresses this issue by using innocuous questions with a known population prevalence (e.g. birthdays) instead of a control group, and embed the sensitive question/statement in a number of innocuous questions (see Table 2). Mathematically, this approach can be described as k innocuous questions with *B(k*n, 0.5)* for the baseline estimation, where *B* is the distribution, k is the number of the innocuous questions in the model and *n* is the sample size. In contrast to previous models such as the Unmatched Count model (Dalton et al., 1994), SSC eliminates the need for a control group and calculates the baseline from personal data with known probability (e.g. birthdays) instead.

An empirical dataset generated via the SSC method contains *n* individual responses where each question in the question set of a number of innocuous questions and one target question is answered with 'yes' or 'no' (true/false in case of a statement format) but only the sum of the answers, which ranges between zero and *k+1*, is recorded. The prevalence rate for the sample is estimated as the difference between the observed average of affirmative responses across the SSC model questions and the expected sum from the *k* innocuous questions, where *d* is the unknown prevalence, *n* is the sample size and *k* is the number of innocuous questions with 50/50 probability (Eq 1). The 95% confidence interval is calculated using normal approximation, where *d* is the estimated proportion of 'yes' answers given to the sensitive target question, *n* is the sample size and Z(0.975) is 1.96 (Eq 2).

$$d = \frac{(number\ of\ yes)}{n} - 0.5k \qquad \text{(Eq 1)}$$

$$(number\ of\ yes) \pm Z(0.975) \times \sqrt{\left(n \times \left(1 + d \times (1 - d)\right)\right)} \qquad \text{(Eq 2)}$$

Take, for example, an SSC model with three innocuous questions with 0.5 probability each and one sensitive target question about doping. Study participants respond with the total number of statements that are true for them (0, 1, 2, 3, 4 or 5), which can be summed for the entire sample. This observed number of 'affirmative' answers is derived from the sum of two random variables: innocuous items such as birthdays or some random numbers, and the sensitive target question with distribution of *B*(*k*n*, 0.5) and *B*(*n*, *d*) respectively, where *d* is the population distribution of doping use and *n* is the number of respondents in the sample. The distribution of the sum of *d* and *k* is unknown, but we can

use the normal approximation for a binomial distribution derived as mean = $n*p$ and variance = $n*p*(1-p)$, where *n* and *p* are the distribution of the two binomial parameters (Petróczi et al., 2011). Illustrating how the SSC works with a numerical example, let us take 1,000 completed surveys where the sum of 'yes' answers is 1,750, giving a mean of 1.75 for the entire sample. We know that the probability from the four innocuous question is 1.5 (= 3 * 0.5). We are interested in the unknown probability of the sensitive target question (e.g. doping use) which we can calculate by taking the difference between the observed mean of 'yes' answers (1.75) and the expected mean from the innocuous questions with 0.5 probability (1.5). The difference is 0.25 for the sensitive target question about doping which is translated as 25% estimated prevalence for admitted doping use.

We used this illustrative example for its simplicity, but the weakness of this specific model setup is a potential exposure in situations where the respondents' have all affirmative answers on the innocuous questions. With a 'yes' on the sensitive target question, one must respond honestly with the maximum of affirmative answers (i.e. '5 yes' answers) which removes the protection for the individual. Although the likelihood for exposure is relatively low, for example 1/16 with a 5-question model, avoiding any exposure is the preferred option. To avoid potential exposure, respondents must be instructed to select a given number of yes answers (i.e. zero) or select any number of yes answers between and including zero to four if their true answer is five. Through a series of simulations, we have shown that from the numerical point of view, there is no difference between the 'select zero' and the 'select any number' variations (Petróczi et al., 2011). For practical reasons, we opted for the 'select zero' variation, presented as a shared button labelled '0 or 5'. The 'zero or 5' model set-up, on one hand rendered the simple equation presented in Petróczi et al. (2011) unsuitable. On the other hand, it offered an option to check for evidence of noncompliance. The suitable alternative approaches are described below in the 'data analysis' section.

With the '0 *or* 5' response option, the simple calculation presented in Petróczi et al. (2011) is no longer suitable. Instead, we used an alternative method with a Maximum Likelihood Estimator (MLE). The method was developed with doping prevalence data in 2012 but owing to the embargo on publication, we validated the method with an independent dataset in 2013 and have made further improvements since (Nepusz et al., 2014). Detecting noncompliance in the SSC with the '0 or 5' response option is straightforward. Here, *p* of 0 is 0.0625 * (*1-d*), and the *p* of 5 is 0.0625 * *d.* Hence, *p* of '0 or 5' is irrespective of *d,* thus *p* of '0 or 5' is 1/16 (6.25%). The significant difference between the observed *p* and the expected *p* = 0.0625 is the evidence for noncompliance. Owing to the model design, we have more data to work with (which means sufficient degrees of freedom, *df*) to take noncompliance into consideration, and to do so without experimental manipulation The advantage of the SSC with a "0 and 5 response" with MLE is that the MLE estimates the probability of the target attribute (e.g. admitted doping use) and the probability of noncompliance, then attributes the noncompliance rate to the proportion of positive (doping) and negative (no doping) cases according to the estimated prevalence rate.

To take 'noncompliance' into account, we need to make assumptions about how this may happen and what the relationship is between noncompliance with the survey instructions and being guilty of doping. The following hypotheses outline six theoretical possibilities with the *null hypothesis* representing the scenario where there is no doping use ($d = 0$) and everyone is compliant with the survey ($c = 0$). *Hypothesis 1* describes the case where doping prevalence is *d* and everyone responds honestly ($c = 0$). In *Hypothesis 2,* doping prevalence is *d* and some respondents are noncompliant by choosing a random answer. In this hypothesis, each respondent may give an honest answer with probability 1-*c* or decide to give a completely random answer with probability *c*. *Hypothesis 3* assumed that doping prevalence is *d* and some respondents choose '0 or 5' with probability *c*. In *Hypothesis 4,* doping prevalence is *d* and respondents select randomly from the lower half ('0 or 5', '1' or '2') with probability *c*. *Hypothesis 5* presents an unlikely scenario where doping prevalence is *d* and some respondents selects '4' or '5' with probability *c*. *Hypothesis 6* defines a case where doping prevalence

is *d* and some respondents select the option that is one affirmative answer less than the truth. This scenario is equivalent to answering only the four innocuous questions and not answering the target question. Nepusz et al. (2014) note that this hypothesis can be equivalent to H1. In practice, it means that the maximum log-likelihood for H6 never exceeds those from the other Hs, whereas the fit will be higher because of the increased complexity. Consequently, this model - although intuitively attractive - cannot mathematically outperform the other hypotheses.

The initial MLE model (Nepusz et al, 2014) assumed that the target attribute and noncompliance are statistically independent. This assumption means that noncompliance can occur with equal probability among those with the target attribute (e.g. doping users) and those without the target attribute (e.g. clean athletes). Although dependent models can also be tested, we have shown in Nepusz et al. (2014) that there is always a solution where dependent and independent models fit equally well. Given these limitations, the recommended use of the noncompliance detection is to check for and estimate the magnitude of noncompliance. Independent from the SSC development (Petróczi et al., 2011; Nepusz et al., 2014), an alternative data processing method for SSC data has been proposed (Groenitz, 2014). This method utilises expectation maximization (EM) algorithm instead of maximum likelihood (Nepusz et al., 2014). The EM method has the advantage of being faster and less likely to produce estimates beyond the 0 and 1 spectrum (thus less likely to produce negative *d* values). We tested both methods on the SSC data and they returned identical results up to three decimals (results not shown).

Regarding the SSC model design, a high number of innocuous questions offers a high level of protection for the respondents. However, a high number of innocuous questions increases the confidence interval (which translates to a less precise estimate), and it is often practically unfeasible due to the limited permutations one can generate from birthdays, and the increase in cognitive load. Therefore, on balance, it is recommended that the SSC model uses three to five innocuous questions/statements.
