# Supplementary material for: Hidden figures: Revisiting doping prevalence estimates previously reported for two major international sport events in the context of further empirical evidence and the extant literature
Source: Front Sports Act Living. 2022 Dec 5;4:1017329. doi: 10.3389/fspor.2022.1017329 (PMC9760848; doi:10.3389/fspor.2022.1017329)
Supplement: Supplementary file 2 [file Data_Sheet_2.pdf]

Supplementary Material 2: Detailed prevalence and noncompliance estimations

Supplementary Table 2.1: Detailed prevalence and noncompliance estimations for WAC and PAG, 2011

Model			WAC doping use	PAG doping use	PAG nutritional supplement use
**Estimation**	***d***	Lowest	0.1251	0.0290	0.0103
		Highest	0.2997	0.1836	0.1611
		Midpoint of multiplied CIs	0.2124	0.1063	0.0857
	CI(d)		0.0872	0.0773	0.0754
	nc	Lowest	0.2628	0.0927	0.0531
		Highest	0.3752	0.1043	0.1755
		Midpoint of multiplied CIs	0.3190	0.0985	0.1143
	CI(nc)		0.0562	0.0058	0.0612
**D**	***d*nc***	Lowest	0.03287628	0.00268714	0.00054693
		Highest	0.11244744	0.019156824	0.02827305
		Midpoint of multiplied CIs	0.07266186	0.010921982	0.01440999
	d*(1-nc)	Lowest	0.07816248	0.02597414	0.00849235
		Highest	0.22093884	0.166587624	0.15254559
		Midpoint of multiplied CIs	0.14955066	0.096280882	0.08051897
**NC**	***(1-d)*nc***	Lowest	0.18403884	0.075647624	0.04454559
		Highest	0.32826248	0.101314104	0.17369235
		Midpoint of multiplied CIs	0.25615066	0.088480882	0.10911897
	(1-d)*(1-nc)	Lowest	0.43754744	0.731216824	0.69167305
		Highest	0.64497628	0.881027140	0.93714693
		Midpoint of multiplied CIs	0.54126186	0.806121982	0.81440999
