# Supplementary material for: Hidden figures: Revisiting doping prevalence estimates previously reported for two major international sport events in the context of further empirical evidence and the extant literature
Source: Front Sports Act Living. 2022 Dec 5;4:1017329. doi: 10.3389/fspor.2022.1017329 (PMC9760848; doi:10.3389/fspor.2022.1017329)
Supplement: Supplementary file 3 [file Data_Sheet_3.pdf]

Supplementary Material 3: Parameters estimates for reliability

Supplementary Table 3.1: Average parameter estimates from resampling for the estimated use of prohibited performance enhancing substances and/or methods at WAC (12-month prevalence)

	**Model fit**			**Estimated % of admitted doping (*d*)**	**Estimated % of noncompliance (*nc*)** p = 8.0E $^{-11}$
**H**	**Log Likelihood**	**AIC**	**BIC**		
H0	-1854.0342	3708.07	3708.07		
H1	-1853.9325	3709.86	3714.96	0.0147	0
H2	-1819.0676	3642.14	3652.32	0	0.3036
H3	-1819.5124	3643.02	3653.21	0.0149	0.0696
**H4**	**-1799.2658**	**3602.53**	**3612.72**	**0.2133**	**0.3182**
H5	-1853.9324	3711.86	3722.05	0.0147	0

Supplementary Table 3.2: Average parameter estimates from resampling for the estimated use of prohibited performance enhancing substances and/or methods at PAG (12-month prevalence)

	**Model fit**			**Estimated % of admitted doping (*d*)**	**Estimated % of noncompliance (*nc*)** p = 0.0394
**H**	**Log Likelihood**	**AIC**	**BIC**		
H0	-1419.4487	2838.90	2838.90	-	-
H1	-1418.2603	2840.52	2843.38	0.0527	0
H2	-1410.8824	2825.77	2835.49	1.4E -5	0.1692
H3	-1415.7747	2835.55	2845.27	0.0514	0.0195
**H4**	**-1413.1278**	**2830.26**	**2839.98**	**0.1065**	**0.0987**
H5	-1418.2612	2840.52	2850.24	0.0517	4.3E -5

Supplementary Table 3.3: Average parameter estimates from resampling for the estimated use of herbal, mineral and/or vitamin supplements at PAG (12-month prevalence)

	**Model fit**			**Estimated % of admitted supplement use (*d*)**	**Estimated % of noncompliance (*nc*)** p = 0.0504
**H**	**Log Likelihood**	**AIC**	**BIC**		
H0	-1405.6629	2811.33	2811.33	-	-
H1/H6	-1405.2946	2812.59	2817.45	0.0282	0
H2	-1398.6209	2801.24	2810.96	0	0.1498
H3	-1403.0769	2810.15	2819.88	0.0288	0.0184
**H4**	**-1398.8987**	**2801.80**	**2811.52**	**0.0862**	**0.1136**
H5	-1405.2347	2814.47	2824.19	0.0093	0.0172
